# Quantifying evolutionary changes to temperature-CO_2_ growth response surfaces in *Skeletonema marinoi* after adaptation to extreme conditions

**DOI:** 10.1093/ismeco/ycaf069

**Published:** 2025-04-18

**Authors:** Charlotte L Briddon, Maria Nicoară, Adriana Hegedűs, Mridul K Thomas, Bogdan Drugă

**Affiliations:** GIMM - Gulbenkian Institute, R. Q.ta Grande 6 2780, Oeiras, Portugal; Institute of Biological Research Cluj, National Institute of Research and Development for Biological Sciences, 48 Republicii Street, Cluj-Napoca, Cluj County 400015, Romania; Institute of Biological Research Cluj, National Institute of Research and Development for Biological Sciences, 48 Republicii Street, Cluj-Napoca, Cluj County 400015, Romania; Doctoral School of Integrative Biology, Faculty of Biology and Geology, Babeş-Bolyai University, 44 Republicii Street, 400015, Cluj-Napoca, Romania; Institute of Biological Research Cluj, National Institute of Research and Development for Biological Sciences, 48 Republicii Street, Cluj-Napoca, Cluj County 400015, Romania; Department of F.-A. Forel for Environmental and Aquatic Sciences and Institute for Environmental Sciences, University of Geneva, 1211 Geneva, Switzerland; Institute of Biological Research Cluj, National Institute of Research and Development for Biological Sciences, 48 Republicii Street, Cluj-Napoca, Cluj County 400015, Romania

**Keywords:** diatoms, generalized additive models, adaptation, trade-offs, response surfaces

## Abstract

Global warming and ocean acidification are having an unprecedented impact on marine ecosystems, yet we do not yet know how phytoplankton will respond to simultaneous changes in multiple drivers. To better comprehend the combined impact of oceanic warming and acidification, we experimentally estimated how evolution shifted the temperature-CO_2_ growth response surfaces of two strains of *Skeletonema marinoi* that were each previously adapted to four different temperature × CO_2_ combinations. These adapted strains were then grown under a factorial combination of five temperatures and five CO_2_ concentrations to capture the temperature-CO_2_ response surfaces for their unacclimated growth rates. The development of the first complete temperature-CO_2_ response surfaces showed the optimal CO_2_ concentration for growth to be substantially higher than expected future CO_2_ levels (~6000 ppm). There was minimal variation in the optimal CO_2_ concentration across the tested temperatures, suggesting that temperature will have a greater influence on growth rates compared to enhanced CO_2_. Optimal temperature did not show a unimodal response to CO_2_, either due to the lack of acclimation or the highly efficient CO_2_ concentrating mechanisms, which diatoms (e.g. *Skeletonema*) can up-/downregulate depending on the CO_2_ conditions. We also found that both strains showed evidence of evolutionary shifts as a result of adaptation to temperature and CO_2_. The evolutionary response differed between strains, underscoring how genetic differences (perhaps related to historical regimes) can impact phytoplankton performance. Understanding how a dominant algal species responds to multiple drivers provides insight into real-world scenarios and helps construct theoretical predictions of environmental change.

## Introduction

Climate change, specifically, global warming and ocean acidification, are a substantial threat to oceanic systems, leading to marine phytoplankton having to adapt to unprecedented rates of change [1]. Oceanic temperatures have increased by ~0.4°C since the 1950s and are projected to increase by an additional 0.5–2°C by the end of the century (RCP4.5 [2]). Concordantly, anthropogenic CO_2_ emissions have led to an average pH decline of 0.1 units since 1850 [3] and are expected to cause a further reduction in the mean surface pH of 0.3–0.4 units by 2100 [4, 5]. The effects of temperature and ocean acidification have been extensively studied on marine ecosystems [6], yet there has been limited work on the evolutionary response of phytoplankton, especially to simultaneous changes in temperature and acidification.

Predicting the effects of multiple drivers is difficult due to their complex interactions [7]. This is compounded by inaccuracies in projecting the precise future CO_2_ and temperature levels, with uncertainties of several ppm and degrees, which is further amplified, the greater the timespan models attempt to predict [2, 8, 9]. In addition, because of the nonlinear response to temperature and CO_2_ and their likely complex interaction, using gradient designs for developing complete temperature-CO_2_ response surfaces are needed to make robust projections, necessary to understand their reaction to multiple simultaneous drivers [10].

Thermal performance curves (TPCs) are a valuable tool to characterize a species’ response to rising temperatures [11]. Previous studies have shown that the shape of the TPCs, and thus the temperature sensitivity of a population, is context dependent, leading to a higher optimal temperature (*T*_opt_) for populations exposed to higher temperatures [10, 12], highlighting species’ capacity for rapid trait shifts in response to environmental change [11]. Yet, due to the concurrent shifts in CO_2_, there is a need to understand how thermal sensitivity shifts with changing CO_2_ [13], with evidence having shown that the thermal sensitivity of phytoplankton is influenced by ocean acidification. Studies using the coccolithophore *Emiliania huxleyi* found shifts of between 1 and 3°C in *T*_max_ depending on the *p*CO_2_ conditions [10] with strains from different geographical regions having distinct responses [14, 15]. This suggests that the environmental context, and thus local adaptation, shapes the response to changes in multiple environmental drivers [16–18].

There has also been minimal work on how the ecophysiological patterns of diatoms vary under changing temperature and *p*CO_2_ conditions. Experimental evolution has been used to quantify changes in physiological traits (such as growth rates) in response to different drivers [19, 20]. This can increase our limited understanding of the evolutionary response of phytoplankton to climatic change [21], with large standing diversity within populations shown to enhance the potential for trait variation within marine ecosystems [19]. To understand the evolutionary response of phytoplankton to climatic change, more precisely to temperature changes and acidification, we conducted an evolution experiment using diatoms that were adapted to different CO_2_ and temperature levels (up to 1% CO_2_; see Table 1 for a list of terminology definitions) before exposing them to a gradient of temperatures and CO_2_ levels. This gradient design allows us to capture the range of shapes the temperature-CO_2_ response surface of a species can take, aiding in the parameterization of models used to predict performance in future environmental conditions.

**Table 1:** Definitions of terminology used in this manuscript.

Acclimation	A slow, reversible process in which an individual organism or population adjusts (through changes in expression of inherited traits) to a change in its environment
Adaptation	Changes in inherited physiological traits (such as growth rates) acquired through generations of evolution to help a population live in a new environment
Evolution	Changes in the heritable traits of a population of organisms as successive generations replace one another
Derived populations	A population obtained through adaptation to different temperature and CO_2_ treatments. These populations have (most likely) evolved as a result of the changes in the growth rates

To get a better picture of how the concomitant changes (interactions) of oceanic warming and acidification will influence phytoplankton responses in future marine ecosystems, we used two strains of the dominant diatom *Skeletonema marinoi* derived from different environments. *Skeletonema marinoi* is an important primary producer, which dominates in coastal regions of temperate oceans [22]. Oceanic warming and acidification have the potential to drive plastic and eco-evolutionary changes within taxa such as *Skeletonema* [23]. Investigating how evolved populations of a dominant species such as *S. marinoi* adapt to projected future conditions (temperature and CO_2_ concentrations) would help us better predict the impacts of these environmental changes on natural populations [24]. Canesi *et al*. [25] found that the relative abundance of different *Skeletonema* species is strongly correlated with temperature, suggesting that this genus is characterized by differentiated thermal traits. Therefore, by analysing the response of two strains of *S. marinoi* from different geographical regions, it is possible to understand if local phenotypic plasticity plays a role in determining thermal trait variation [26]. This would allow us to draw conclusions about the thermal limits of different populations and for more reliable projections to be made into how marine ecosystems will respond to future changes in multiple environmental dimensions [27].

The aim of this study was to quantify the response surfaces along gradient of temperature and CO_2_ for two strains of *S. marinoi* and to determine how our results match with our predictions (Table 2). We used a 5 × 5 factorial experimental design resulting in 25 populations grown under a combination of temperatures and CO_2_ concentrations. We aim to use our results to help answer a number of unknown questions (Table 2) including (1) how does the optimal CO_2_ concentration change with temperature and (2) are there any constraints or trade-offs that we can identify? Understanding the response of a dominant algal species to multiple drivers provides usable insights into real-world phenomena and helps to inspire theoretical frameworks to predict the response to environmental change.

**Table 2:** Testing hypotheses and unknown questions.

Prediction	Supported?	Figure
*T*_opt_ should increase as a result of adaptation to higher temperature, at the CO_2_ level at which the strains were adapted	Yes/No. S8 demonstrates a shift in *T*_opt_, with a greater shift observed in the samples adapted to 19°C rather than 13°C, but this did not occur consistently in strain S16	1, 2, Supplementary
*T*_opt_ should be a unimodal function of CO_2_	Yes/No. Some of the lower temperature (13°C, 16°C) demonstrated a unimodal response to CO_2_ in the strains adapted to 13°C and to both CO_2_ conditions	2, Supplementary
Adaptation should result in an increase in growth when tested in the same conditions in which they were adapted to for both temperature and CO_2_, e.g. samples adapted to 25°C should demonstrate a higher growth rate when tested in that temperature (when using the same CO_2_ concentration of adaptation)	Yes/No. S16 demonstrated an increase in peak growth rates when tested back in the same adaptation conditions, whilst S8 demonstrated limited differences in growth rates	2
Unknowns		
How does optimum CO_2_ [assuming one exists in these curves] change with temperature? How will the different historical temperature regimes experienced by the two strains affect their evolutionary response? How does adaptation to different temperature and CO_2_ conditions change the shape of the response surface along a range of high temperature and unrealistic CO_2_ conditions? Are there evolutionary costs or trade-offs associated with adaptation to temperature and/or CO_2_?		

## Materials and methods

### Biological materials and growth conditions of the *Skeletonema* strains (S8 and S16)

Two strains of *S. marinoi* (S8 and S16) were isolated from water samples collected from two locations along the Norwegian Coast. S16 was isolated from southern Norway (temperature at the time of sampling: 13.4°C and 25.5 psu), specifically, Sognesjøen (61.1554° N, 6.5806° E; average yearly water temperature of 10.7°C), in the Sognefjorden region on the Norwegian West Coast. S8 was collected from the outer part of Tanafjorden, Northern Norway (70.8306° N, 28.4723° E; average yearly water temperature of 8.1°C; temperature at the time of sampling: 10.1°C and 33.86 psu). For details on the deposition of both strains in the Collection of Cyanobacteria and Algae (AICB) at the Institute of Biological Research in Cluj-Napoca, Romania [28], see [23]. The S16 and S8 strains were assigned to *S. marinoi* via sequencing of 18S rRNA gene amplified with primers designed by [29] by a third-party company (Macrogen Europe, Amsterdam, The Netherlands). The sequences have been deposited in GenBank under the accession numbers PP600221 (S8) and PP600222 (S16).

### Evolution experiment

The S8 and S16 strains were exposed to two different temperature conditions (13°C and 19°C) for 15 months (August 2021 to November 2022; ~300 generations) and two *p*CO_2_ conditions (400 and 1000 μatm) for 12 months (November 2021 to November 2022; ~240 generations). We chose these two different *p*CO_2_ conditions based on the present versus year 2100 *p*CO_2_ levels and the projected temperatures for 2050 and 2100 under scenarios SSP5–8.5 and SSP2–4.5 from the latest IPCC climate model output (CMIP6; [2]) for both sample collection sites (Tanafjorden and Sognesjøen, Norway). This is consistent with multiple studies which have used these two selection environments to explore how ocean acidification will influence marine phytoplankton [30, 31]. Three replicates were used for each strain (which we term ‘evolutionary/derived replicates’ hereafter) resulting in four strain × CO_2_ × temperature combinations for each strain (Fig. S1). The postponement with exposing the strains to the different *p*CO_2_ conditions was due to a delay in obtaining the gas tanks with the required *p*CO_2_ concentrations. An initial thermal performance curve completed on both strains showed that the *T*_opt_ for S16 was ~20°C, whilst for S8 it was ~18°C. The temperature of 13°C was also chosen as it was a relevant temperature for both strains given their different historical temperature regimes from their sampling sites.

To acclimate the strains to different *p*CO_2_ concentrations, each sample was bubbled with artificial air containing either 400 ppm or 1000 ppm CO_2_ (Messer, Bad Soden, Germany) for 15 min every 3 h (120 min per day). The frequent bubbling was also necessary to prevent cell clumping. The strains were maintained in semi-batch conditions in artificial seawater (f/2 medium [32]) with added macronutrients [33] and micronutrients/trace metals [34]. For the full list of macro- and micronutrients added, see the supplementary material of [23]. Samples were grown in 100-ml glass tubes under controlled 16 h:8 h light/dark conditions provided by white LED lamps (100 μmol photon m^−2^ s^−1^; [35–37]). During this entire experiment, the strains were maintained as semi-batch cultures: they were grown until reaching an optical density OD600 = 0.5–0.6, and then they were transferred to a new medium. Following the long-term selection exposure experiment, all derived strains were exposed to five temperatures and five *p*CO_2_ conditions as shown in the experiment schematic (Fig. S2).

### Response surface experiment

We measured the unacclimated growth response surfaces of both strains (S8 and S16) that were previously exposed to selection at all four temperature × CO_2_ combinations (13°C and 400 ppm; 19°C and 400 ppm; 13°C and 1000 ppm; 19°C and 1000 ppm, with three evolutionary replicates per treatment). All the derived populations were tested using a 5 × 5 factorial experimental design consisting of five temperatures (13°C, 16°C, 19°C, 22°C, and 25°C) and five CO_2_ conditions (400 ppm (pH 8.13–8.17), 1000 ppm (pH 7.77–7.82), 2500 ppm (pH 7.36–7.43), 5000 ppm (pH 7.09–7.15), and 10 000 ppm (pH 6.79–6.85); Fig. S2). All pH values are on NBS (National Bureau of Standards) scale. We used these ‘unrealistic’ CO_2_ conditions to better understand the shape of the temperature-CO_2_ response surface across a very broad range of conditions and motivate the development of better models of growth dependence on temperature and CO_2_ [8]. Additionally, realistic response surfaces have shown that moderate uncertainty in environmental predictions can lead to entirely different biological outcomes, so our use of extreme CO_2_ levels allows us to take this into account. For each tested condition, the CO_2_ and DIC levels are stated in Table S1. Alkalinity was kept constant at between 2290 and 2310. The strains were not acclimated to each condition prior to beginning each of the response surface experiments or for the initial TPCs. Although this would have been ideal, given that it would enable comparison with published response curves and surfaces that tend to use acclimated growth rates, acclimating pre-exposed cultures to the extreme CO_2_ and temperature levels used in this experiment was unfeasible due to logistical issues. Consequently, we proceeded directly to testing without prior acclimation. This approach may capture immediate physiological responses alongside evolutionary changes, which is common in phytoplankton evolution studies where sustained physiological acclimation is difficult to separate [38, 39]. Due to the number of treatments, we could not run all samples concurrently. Therefore, we started off with the 400-ppm, followed by the 1000-ppm, 5000-ppm, 10 000-ppm, and finally the 2500-ppm experiment. The experiments were completed in 100-ml glass tubes using the same procedure as during the long-term exposure process, with the cultures being bubbled with one of the CO_2_ conditions for 2 weeks to test their response to each CO_2_ treatment. Before each experiment, the *S. marinoi* strains were diluted to the same starting optical density (OD_600_ = 0.05; ~1.06 × 10^3^ cells ml^−1^). On Days 2, 4, 7, 9, 11, and 14, the OD was measured using 3 ml collected from each test tube (and then subsequently disposed of) to determine the growth rates. If the sample died, further OD measurements were not taken, and the OD was recorded as zero. We used optical density (OD) as a proxy for growth rather than cell counts or fluorescence measurements consistent with other phytoplankton studies [40–42]. *Skeletonema marinoi* is a filamentous microalga, making precise cell counts challenging [43]. Fluorescence measurements, whilst feasible, would have required at least 4 min per sample using our PhytoPAM II device, which was impractical given the large number of parallel samples.

### Calculating the specific growth rate

Specific growth rates were calculated for each evolutionary replicate using the get.growth.rate function (growthTools package ([44]; R, version 4.3.1 [45]). First, we fitted five models to the measured time series of density for each evolutionary replicate. The models were as follows: a linear model (‘linear’); a smoothed piecewise linear model (‘lag’) to ln(optical density) data, with the assumption that abundances are nearly constant for several time points, before exponential growth begins; a smoothed piecewise linear model (‘sat’) to ln(abundance) data, with the assumption that abundances increase linearly at first, but then saturate and remain constant; a smoothed piecewise linear model (‘flr’) to ln(abundance) data, with the assumption that abundances decrease linearly at first, but then hit a floor and remain constant; and a smoothed piecewise linear model (‘lagsat’) to ln(abundance) data, with the assumption that abundances are nearly constant for several time points, before exponential growth kicks in—subsequently, growth saturates and abundances become constant again. As these models can only be determined using monotonic data, any datapoints collected during the death phase had to be filtered out prior to analysis. Second, we utilized the AICc (Akaike information criterion) to determine the best-fitting models for each evolutionary replicate. We found that a linear model resulted in the best fit but as the log-OD plateaued, indicating that the *S. marinoi* strains reached the stationary growth phase during the 2-week experiment. Therefore, we calculated the growth rates only using the period where the growth rate was exponential (Days 0–4 to ensure consistency across all samples). The slope of the resulting linear regression was the specific growth rate (*μ*) of each replicate.

### Determining variation in specific growth rates using generalized additive models

We used generalized additive models (GAMs) to characterize variation in the response surfaces as we do not presently have a parametric model that is able to [8, 46, 47]. Both the tested conditions (temperature and the CO_2_ concentration) were used as smoother terms, with tensor smoothing products, which consider the different units of the independent terms and define the vector space of the temperature*CO_2_ with a basis spanning the product of both variables. Curvature of the GAM-interpolated contours showcased how growth rates changed with temperature and CO_2_. This analysis was completed using ‘mgcv’ package in R (version 4.3.1).

### Thermal performance curves

The following function was used to determine the temperature response of growth rates for all strains across all CO_2_ treatments including for the strains initially (e.g. pre-selection) and the evolved stains: $\mu (T)={ae}^{bT\left[1-{\left(\frac{T-z}{w/2}\right)}^2\right]}$, where the specific growth rate (*μ*) is a function of temperature (*T*). The species traits (*z* and *w*) controls the shape of the temperature function curve. *w* is the thermal niche width, whilst *z* decides the position of the location of the maximum of the quadratic portion of this function. *a* and *b* both determine the height, skewness, and steepness of the curve. A parametric bootstrapping approach was used (using a Monte Carlo approach) to create 1000 bootstrapped simulations for all parameter estimates, which were used to calculate the 95% confidence intervals of all parameters, including derived ones such as the optimal temperature (*T*_opt_) and the maximum growth rate. For further information, see [48, 49].

## Results

### Initial thermal performance curves

**Figure 1:**
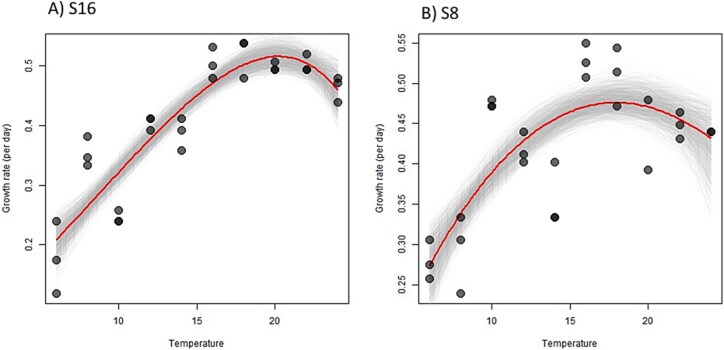
Thermal reaction norms of the two *S. marinoi* strains (A) S16 and (B) S8 taken prior to the start of the adaptation experiment.

We used TPCs completed prior to the beginning of the evolution experiment to understand the impact of 12 plus months of selection and whether it resulted in a shift in the *T*_opt_ and the maximum growth rates. There were differences between the initial TPCs for the two strains (S8 and S16; Fig. 1). Strain S8 had a lower *T*_opt_ of ~18°C compared to S16 which had a *T*_opt_ of ~20°C (Table S2). S16 also had a higher maximum growth rate of 0.52 day^−1^ compared to 0.48 day^−1^ for S8. The comparison of the initial and the evolved TPCs (after 12 months of selection under differing temperature conditions) allowed us to determine how selection influenced growth rates and shifts in thermal properties.

### Determining temperature-CO_2_ effects on growth using GAMs

**Figure 2:**
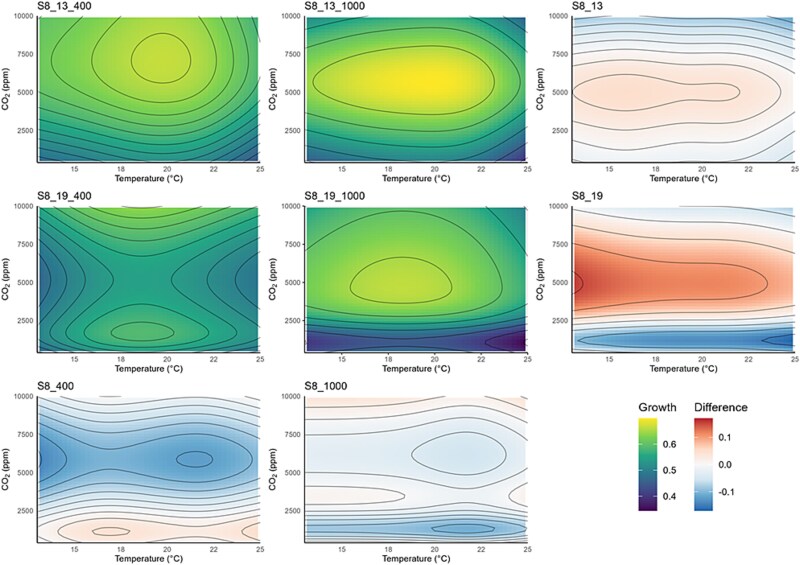
Temperature-CO_2_ response surfaces (calculated using GAMs) showing variation in growth rates across all temperature and CO_2_ conditions for strain S8. S8_13 and S8_19: The evolutionary shifts due to adaptation to different CO_2_ conditions (determined by subtracting the 1000-ppm response surface growth rates from the 400-ppm ones and adding a GAM smoother). S8_400 and S8_1000: The evolutionary shifts due to adaptation to different temperature conditions (determined by subtracting the 19°C response surface growth rates from the 13°C ones and adding a GAM smoother).

**Figure 3:**
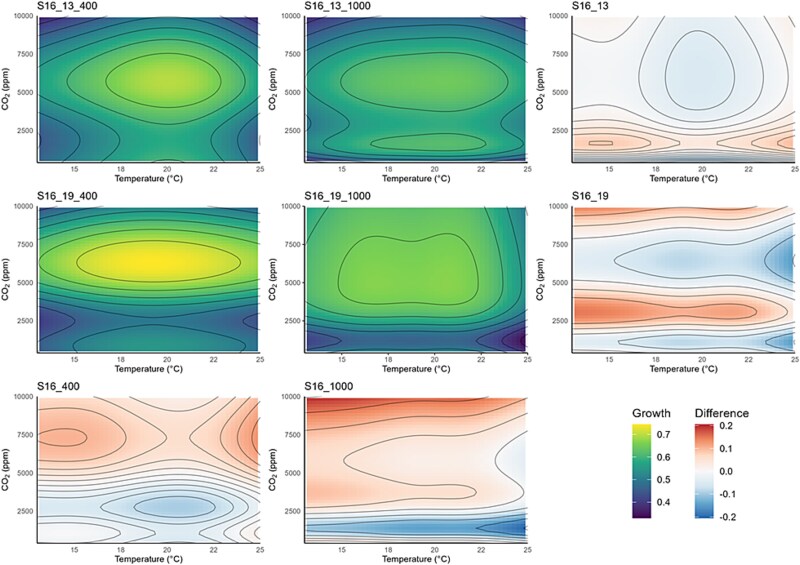
Temperature-CO_2_ response surfaces (calculated using GAMs) showing variation in growth rates across all temperature and CO_2_ conditions for strain S16. S16_13 and S16_19: The evolutionary shifts due to adaptation to different CO_2_ conditions (determined by subtracting the 1000-ppm response surface growth rates from the 400-ppm ones and adding a GAM smoother). S16_400 and S16_1000: The evolutionary shifts due to adaptation to different temperature conditions (determined by subtracting the 19°C response surface growth rates from the 13°C ones and adding a GAM smoother).

The response of the *S. marinoi* strains exposed to different temperature and CO_2_ treatments was highly variable (Fig. 2; Fig. 3; Fig. S3; Fig. S4). The GAMs produced in the 5 × 5 factorial experiment showed broadly similar patterns in growth rate variation (with the one exception being S8 previously exposed to 19°C and 400 ppm; Fig. 2), with the peak growth rate estimated to be ~6000 ppm CO_2_ (Fig. 2). The temperatures at which this peak in growth rate occurred was strain specific but generally occurred in the 16–24°C range. For S8 acclimated to 19°C and 400 ppm, the peak growth rate occurred at >10 000 ppm over a broad range of temperatures (17–22°C; Fig. 2). These results were consistent with the temperature function curves and the thermal response curves at different CO_2_ levels with the peak growth rate occurring between the higher CO_2_ treatments (5000 and 10 000 ppm; Fig. S5d, E; Fig. S6d, E; Fig. S7; Table S3).

The peak growth rate differed between samples and was highest in the S16 acclimated to 19°C and 400 ppm (S16_19_400) and S8_13 (exposed for long term to both CO_2_ conditions) of >0.75 day^−1^, whilst S16_19_400 peaks at 0.7–0.75 day^−1^ and the remainder of the samples at >0.7 day^−1^. At the extremes (for both temperature and CO_2_), the growth rate is depressed for all samples (except being S8 previously grown at 19°C and 400 ppm; S8_19_400) indicated by the green colouring on the contour plots. S16 exposed to 13°C and 1000 ppm had multiple peaks in growth rate on the response curve (Fig. 3), which was not observed in the other samples. This bimodality is biologically implausible and was likely due to noise in the data and consequently a poor GAM fit. There were also no clear differences in the thermal niche width between the CO_2_ treatments, strain, and long-term exposure conditions.

The results suggest that *T*_opt_ demonstrated a unimodal response of CO_2_ for the lower tested temperatures (13°C and 16°C, Fig. S7b); however, this pattern was less clear for the higher tested temperatures (19, 22, and 25°C). The lack of a clear pattern in the higher tested temperatures could be due to noise in the data.

### Evidence of evolutionary shifts

Both strains showed evidence of shifts due to selection exposure (Fig. 2; Fig. 3), although it was not uniform across the response surfaces. The GAM response surfaces showed that these shifts (e.g. where there was greatest change in growth rate) occurred at low CO_2_ treatments (Fig. 2), with a stronger response in S8 compared to S16. For the CO_2_ selection exposure, the greatest shift generally occurred in the lowest CO_2_ treatments closest to the CO_2_ concentrations used during the long-term exposure experiment, with the least amount of change occurring in the central CO_2_ concentrations and temperatures. The exception to this were S16 exposed to 19°C and S8 exposed to 400 ppm which demonstrated the opposite pattern. Moreover, after long-term exposure to different temperatures, we again observed change in the edges of the response surfaces, but also in the central CO_2_ and temperatures tested (especially in S8 strain). S16 demonstrated an increase in peak growth rates when tested in the same temperature conditions of those it was pre-acclimated to, whilst the strain S8 demonstrated limited differences in growth rates. Our results showed that previous exposure to enhanced temperature and CO_2_ did influence growth rates at higher temperatures and/or CO_2_ conditions. However, it did lead to a shift in *T*_opt_ for strain S8 but not for strain S16. S8 demonstrates a shift in *T*_opt_ (Fig. 2), with a greater shift observed in the samples grown at 19°C compared to 13°C, but this pattern was not detected in strain S16 (Fig. 3).

## Discussion

We examined how two strains of *S. marinoi* changed in response to a range of temperature and CO_2_ concentrations to capture the temperature-CO_2_ response surfaces. The use of a 5 × 5 factorial design allowed for the prediction of ecological responses to environmental conditions not measured during this experiment via interpolation and limited extrapolation [50], as well as the individual and interactive effects of temperature and CO_2_ [51]. Our results showed the variable and strain-specific effects of previous exposure on the response surfaces, highlighting the impact that intraspecific variation can have on survival in future conditions [52].

There were similar patterns in growth rate variation across the response surfaces, suggesting that whilst the different selection pressures act in various ways, they achieve a similar outcome. *Skeletonema* (and diatoms in general) possesses highly efficient CO_2_ concentrating mechanisms (CCMs), which allow diatoms to maintain good photosynthetic performance under low CO_2_ conditions [53]. Higher inorganic carbon availability under ocean acidification has also been shown to downregulate CCMs in diatoms resulting in this saved energy being available for carbon fixation and, therefore, growth [54]. However, marine organisms need to use additional energy to maintain their intracellular pH caused by a concurrent pH decrease [55]. Consequently, their efficient CCMs and cellular damage due to high pH could explain the lack of variation in the response in the CO_2_ curves. Diatoms (including the *Skeletonema* genus) have a reduced sensitivity to high *p*CO_2_ compared to other phytoplankton groups and are able to maintain peak growth rates at a wider range of *p*CO_2_ (up to ~7500 ppm [56, 57]). This could also be a possible cause of the peak CO_2_ concentration for growth rate at ~6000 ppm for most samples. Whilst the exact reason for peak growth rates at this CO_2_ concentration is unknown, it could represent the value at which the *S. marinoi* was able to best balance the positive and negative effects of enhanced CO_2_. It is unknown what the reality of a peak growth rate of 6000 ppm would entail in natural conditions. Sett *et al*. [58] demonstrated a decline in metabolic rates in coccolithophores at these very high CO_2_ concentrations, with the negative effects being modulated by higher temperatures. This suggests that even though CO_2_ concentrations of 6000 ppm are not predicted for the next century, the concurrent increase in temperature could modulate some of the negative impacts for phytoplankton, suggesting the potential for marine diatoms to cope with extreme conditions in the short term.

It has been well documented that enhanced temperatures and CO_2_ lead to an increase in growth rate (e.g. unimodal response; [49, 56, 59]), yet the response to both temperature and CO_2_ simultaneously is ambiguous. Previous studies have shown that an increase in temperature caused an enhancement of growth rate at all CO_2_ concentrations in two diatom species (*Thalassiosira* sp. and *Nitzschia closterium* [53]). However, for *S. marinoi*, there was little variation amongst the tested CO_2_ treatments, implying that temperature may have a greater influence on growth rates compared to enhanced CO_2_ for this diatom. This is in conjunction with previous studies that have shown that *S. marinoi* exhibited an increase in growth rates with an enhancement in temperature, whereas the response to increased *p*CO_2_ was strain specific [24] and could depend on the size and shape of the diatom [53]. Larger centric diatoms, like *Skeletonema*, with their smaller surface-to-volume ratio require higher CO_2_ levels to saturate growth compared to pennate diatoms such as *Nitzschia* [60, 61]. However, as cell size measurements were not collected during this experiment, we cannot determine if pre-exposure to different temperatures led to changes in cell size, which may account for some of the strain-specific differences. This suggests that there could be wide intra- and interspecific variability in the response of CO_2_ on diatoms [56] underscoring the dominant influence of temperature on future predictions.

Historical environmental conditions and differences in strain physiology played a substantial role in influencing the evolutionary response of *S. marinoi* to different temperatures and CO_2_ conditions. The strain collected from northern Norway (S8) demonstrated a greater shift in *T*_opt_ when exposed to higher temperatures compared to the strain from southern Norway (S16), consistent with previous research which found that the origin of phytoplankton can influence phytoplankton TPCs [52, 62]. Boyd *et al*. [63] highlighted that adaptation to higher temperatures could help to mitigate the lack of oceanic refugia especially for polar species; however, there is also evidence that other phytoplankton groups (e.g. coccolithophores) could emerge or replace diatoms. Nonetheless, natural communities consist of multiple coexisting strains and selection between them could be a possible source of intraspecific plasticity, potentially mitigating the impacts of climatic change [64].

The long-term exposure to temperature and CO_2_ resulted in a mixed response with respect to growth rates, with some evolutionary replicates experiencing an increase in peak growth when tested back in the same conditions and others not. Kremer *et al*. [65] found that phytoplankton can express a variety of beneficial and detrimental long-term responses, with trade-offs being a possible cause. Selection under high temperatures and CO_2_ resulted in mostly a decline in growth rates at lower temperatures and CO_2_ concentrations, e.g. a performance trade-off [66]; however, this pattern was not universal. Under suboptimal levels of other environmental factors, adaptation to rising temperature may be slowed down due to trade-offs between temperature and resource requirement [27]. Our results support this, as the greatest shifts generally occurred at suboptimal CO_2_ concentrations. It is possible that the differing response to selection exposure with regard to growth rate could be due to investment in costly machinery (such as cellular repair machinery or the production of heat-shock and thermal tolerance proteins) that reduces growth in some samples yet enhances survival at high temperatures [65]. Lauritano *et al*. [67] found that in *S. marinoi*, glycolate oxidase (GOX) genes were activated when exposed to stressful environments (such as CO_2_ enrichment) allowing diatoms to better cope with adverse conditions, whilst other genes such as antioxidant-related and ALDH genes were strongly downregulated, suggesting that cells were avoiding unnecessary investing in these proteins [68]. Some of these observed phenotype changes could be attributed to transgenerational plasticity and/or epigenetic inheritance, where selection under different temperatures caused certain genes to be expressed and maintained across generations due to the ongoing change [69]. Although gene expression was not a part of this study, there remains a possibility that the upregulation or downregulation of certain genes could explain the trade-offs observed, which would be worth exploring in future research.

The lack of acclimation (before starting the 5 × 5 experiment) to the tested conditions would have influenced the growth rates estimated in this study [65, 70] and is a likely cause of noise or unknown biases in the data (e.g. bimodality). This experimental necessity does limit the ability to compare our growth rate estimates with published values that mostly use acclimated cultures. However, it would be interesting to explore the effect of using unacclimated strains to investigate the impact of heatwaves, which has only been studied with respect to copepods [71]. This study found that heatwaves that occur near/past the thermal optimum may decrease performance; this could (partially) explain why at the extremes (for both temperature and CO_2_), the growth rate is depressed for all samples. Wang *et al*. [72] determined that *Microcystis aeruginosa* populations acclimated to temperatures lower than the tested temperature demonstrated enhanced growth, whilst those acclimated to temperatures higher than the tested temperatures exhibited a decline in performance. However, the impact of this is dependent on the severity of diurnal fluctuations [65]. The temperatures in the water baths (in which the experiments were conducted) were kept constant, so the effects of fluctuations would have been minimal. If this prediction is correct, particularly for the samples pre-exposed to 19°C, this could have resulted in a change of the shape of the TPCs, with the left skewed curves possibly shifting further to the left and a dampening of the *T*_max_. Even though the results (including GAMs) should be interpreted with some caution, these results have given us the first complete temperature CO_2_ growth surfaces, allowing us to make improved projections of species’ responses to environmental change, necessary to understand the reaction of *S. marinoi* to multiple simultaneous drivers.

Temperature-CO_2_ response surfaces have the ability to significantly aid our understanding into how species will respond to future conditions and the resulting performance trade-offs; however, other factors need to be considered. These surfaces also need to consider other variables which can influence growth rates and TPCs such as light and nutrient levels [73–76]. Previous research has shown that the interactions between increasing temperature and nutrient limitation would cause a decline in species’ *T*_opt_ and *T*_max_ [77], with possible implications for oceanic primary production. As enhanced stratification (which is linked to warmer temperatures [78]) can cause a reduction of nutrients and increase in light supply [79], it is vital to include additional variables such as light and nutrients to allow for further research into how the interactions between them influence growth rates and help improve the accuracy of predictions.

## Conclusion

In summary, temperature-CO_2_ growth response surfaces will help us to make useful predictions about how drivers interact to influence species, now and under future conditions. We showed that long-term exposure to different CO_2_ and temperature levels can cause evolutionary shifts in *T*_opt_, maximum growth rates, and temperature-CO_2_ response surface shape. Our use of response surfaces of very high CO_2_ allowed us to better constrain the shape of the growth landscapes even in the 400–1000-ppm range [8, 56]. The evolutionary response varied between strains, highlighting the effect of historical regimes on adaptation. Temperature-CO_2_ growth response surfaces can help forecast species responses to predicted environmental change, which can be built on using community experiments. This work can help bridge the gap between theoretical and empirical data to help us understand how changing environments will continue to evolve. Even though this was not the focus on this study, it would be interesting to explore in the future if unacclimated TPCs could be used to explore the effects of future heatwaves on phytoplankton populations.

## Supplementary Material

Supplementary_Information_Briddon_ycaf069

## Data Availability

The data that support the findings of this study are available from the corresponding author upon reasonable request.
