# Supplementary material for: Quantifying evolutionary changes to temperature-CO2 growth response surfaces in Skeletonema marinoi after adaptation to extreme conditions
Source: ISME Commun. 2025 Apr 18;5(1):ycaf069. doi: 10.1093/ismeco/ycaf069 (PMC12075770; doi:10.1093/ismeco/ycaf069)
Supplement: Supplementary_Information_Briddon_ycaf069 [file supplementary_information_briddon_ycaf069.pdf]

Supplementary Information

Title: Quantifying evolutionary changes to temperature-CO_2 growth response surfaces in *Skeletonema marinoi* after adaptation to extreme conditions

Authors: Charlotte L. Briddon, Maria Nicoară, Adrianna Hegedüs, Mridul K. Thomas, Bogdan Drugă

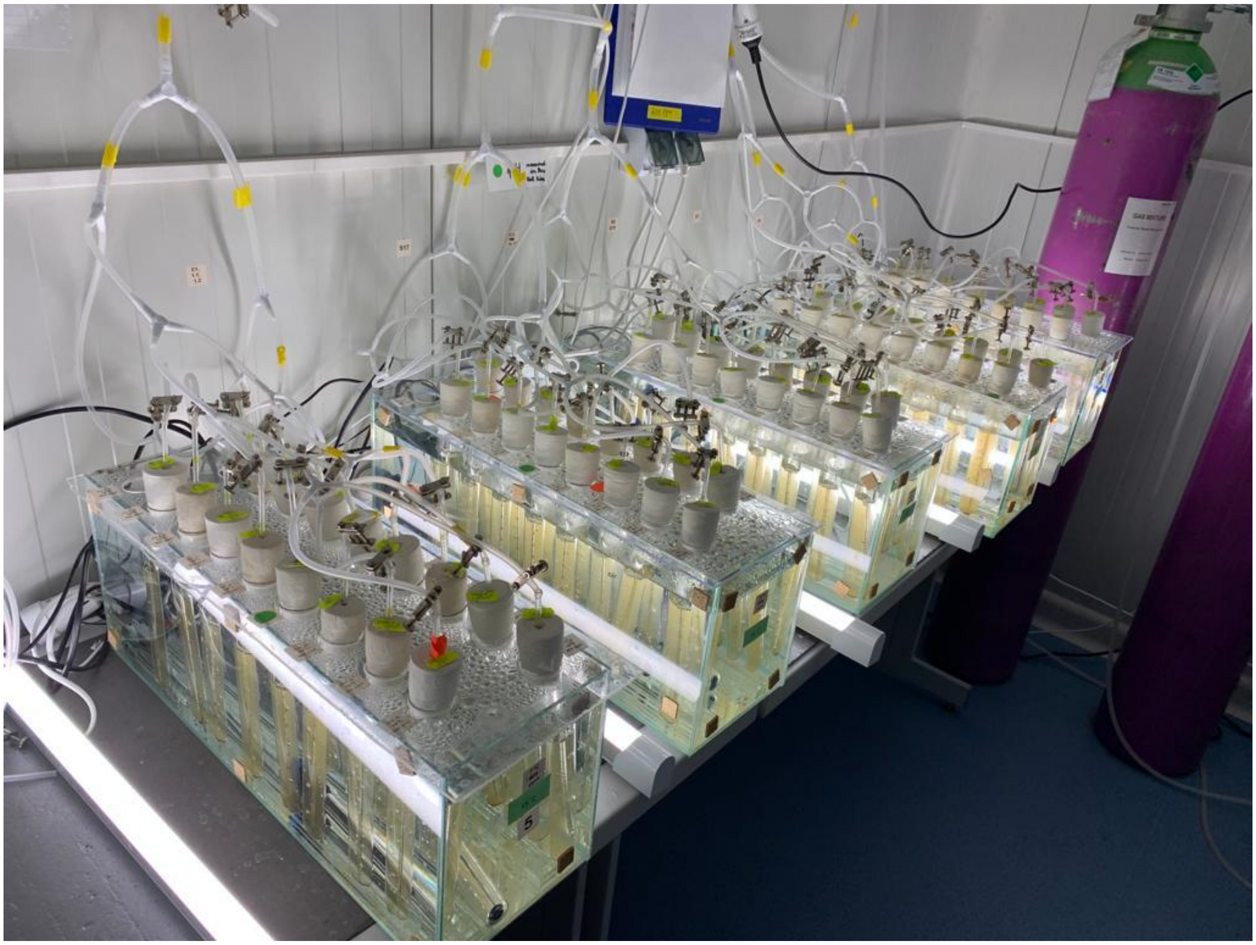

Figure S1: A photograph showing the response surface experiment layout. Each evolutionary replicate is placed in test tube in a water tank heated to the desired temperature (either 13℃, 16℃, 19℃, 22℃ or 25℃). In order to expose the S. marinoi to different CO_2 concentrations, each test tube was bubbled with air from a gas tank with the desired CO_2 concentration (either 400, 1000, 2500, 5000 or 10,000 ppm). The same set-up was used for the evolution experiment.

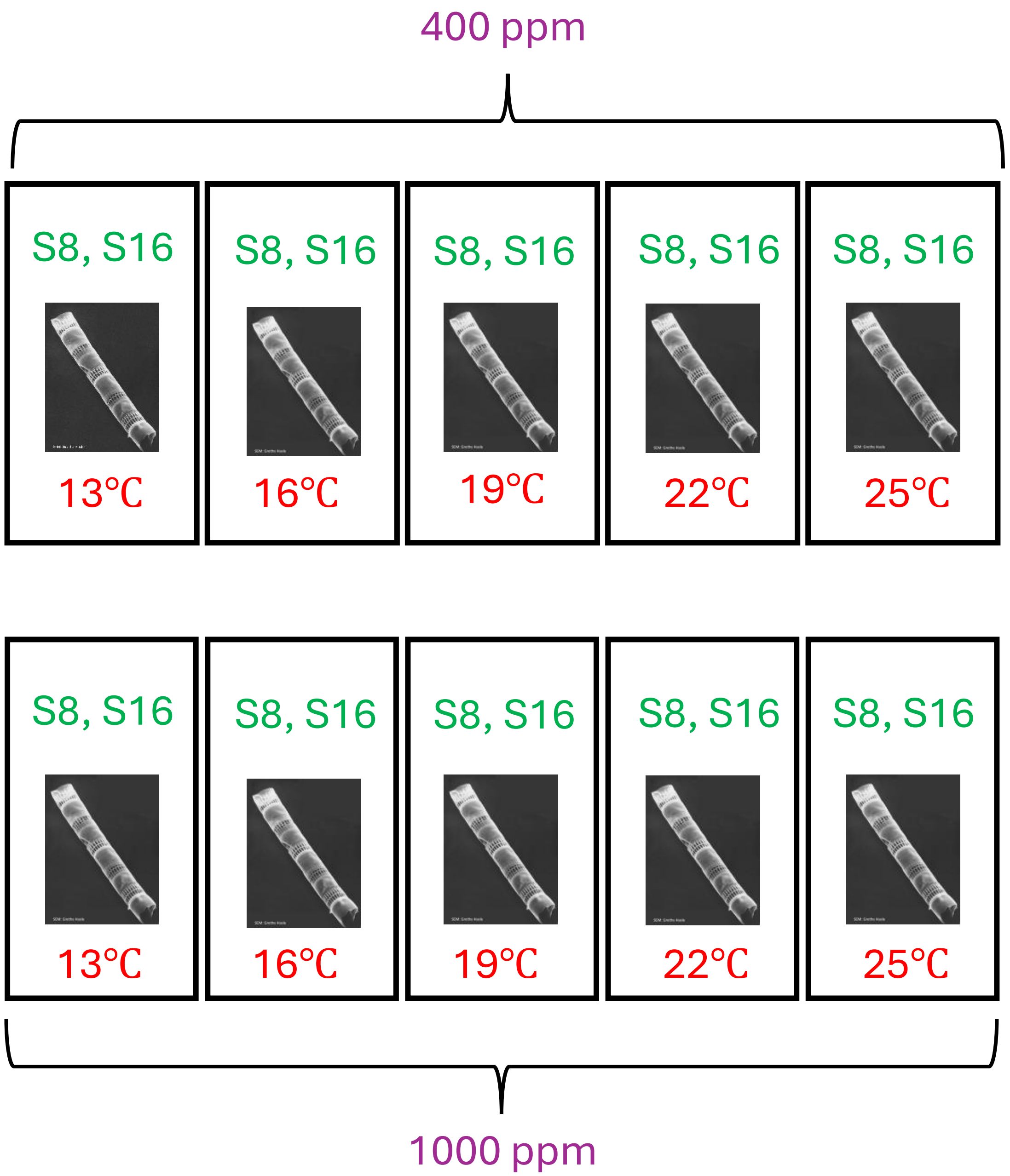

Figure S2: A schematic of the evolution experiment showing how both strains (S8 and S16) were adapted to a combination of temperatures and CO_2 treatments resulting in all strains exposed to all treatments. The black boxes represent the aquariums which contained the S. marinoi strains and were heated to the desired temperature (13℃ or 19℃). In order to adapt the strains to the two different CO_2 treatments (400 and 1000 ppm), all samples were bubbled with desired CO_2 concentration. Overall, ten aquaria were used to adapt the samples. The same layout was used for the response surface experiment but with additional CO_2 treatments (2500, 5000 or 10,000 ppm). The temperature was controlled using the same manner.

Table S1: The actual CO_2 and dissolved inorganic carbon (DIC) concentrations for each tested temperature (13℃, 16℃, 19℃, 22℃, 25℃) and CO_2 (400, 1000, 2500, 5000, 10000 ppm) condition used in the response surface experiment				
Temperature (℃)	pCO_2	TA in (mmol/kgSW)	TCO_2 in (mmol/kgSW); DIC	pH
13	400	2300	2100.7	8.15
15	400	2300	2088.4	8.15
18	400	2300	2069.3	8.15
22	400	2300	2042.6	8.15
25	400	2300	2021.6	8.15
13	1000	2300	2234.0	7.80
15	1000	2300	2226.2	7.80
18	1000	2300	2214.1	7.80
22	1000	2300	2197.5	7.80
25	1000	2300	2184.5	7.80
13	2500	2300	2361.1	7.40
15	2500	2300	2354.2	7.40
18	2500	2300	2344.3	7.40
22	2500	2300	2331.7	7.40
25	2500	2300	2322.4	7.40
13	5000	2300	2481.2	7.12
15	5000	2300	2471.9	7.12
18	5000	2300	2459.0	7.12
22	5000	2300	2443.4	7.12
25	5000	2300	2432.8	7.12
13	10000	2300	2701.6	6.82
15	10000	2300	2685.3	6.82
18	10000	2300	2663.2	6.82
22	10000	2300	2637.4	6.82
25	10000	2300	2620.4	6.82

Table S2: The confidence intervals (CI) from the 1000 bootstrapped simulations for the optimal temperature from both strains (S8 and S16) pre-adaptation			
**Strain**	**Optimal Temperature**	**Lower CI**	**Upper CI**
S8	17.92522	15.6849	5419.371
S16	20.19293	19.0461	4436.269

Table S3: Model parameters of the thermal performance curves and how they vary by strain, CO_2, and evolution treatment.<br>*Erroneous value					
Sample	Temp_tested (℃)	CO_2_tested (ppm)	CO_2 Treatment (ppm)	Optimum Temperature	Maximum Growth Rate
S16_13_1000	13	1000	400	20.56714022	0.484723815
S16_13_400	13	400	400	20.21240186	0.56600177
S16_19_1000	19	1000	400	15.29972295	0.533375937
S16_19_400	19	400	400	13.91469936	0.545907264
S8_13_1000	13	1000	400	17.15935164	0.490929459
S8_13_400	13	400	400	16.9362426	0.490856701
S8_19_1000	19	1000	400	15.68732387	0.497942099
S8_19_400	19	400	400	15.55508709	0.531230536
S16_13_1000	13	1000	1000	11.55810914	0.585695961
S16_13_400	13	400	1000	18.90337745	0.569318124
S16_19_1000	19	1000	1000	17.38943586	0.520254629
S16_19_400	19	400	1000	17.25717697	0.5361704
S8_13_1000	13	1000	1000	16.17686203	0.540877797
S8_13_400	13	400	1000	-6.94581145*	0.582149009
S8_19_1000	19	1000	1000	16.62455177	0.448238429
S8_19_400	19	400	1000	15.07827227	0.590349893
S16_13_1000	13	1000	2500	16.62126289	0.566786041
S16_13_400	13	400	2500	19.34664281	0.59127396
S16_19_1000	19	1000	2500	19.14156936	0.604745833
S16_19_400	19	400	2500	18.00947681	0.529684168
S8_13_1000	13	1000	2500	14.72521391	0.567404645
S8_13_400	13	400	2500	19.19125845	0.581650947
S8_19_1000	19	1000	2500	20.22233838	0.563427036
S8_19_400	19	400	2500	18.90323374	0.581490203
S16_13_1000	13	1000	5000	18.98861615	0.655940545
S16_13_400	13	400	5000	21.07800269	0.688370325
S16_19_1000	19	1000	5000	18.25759115	0.690111561
S16_19_400	19	400	5000	20.70169156	0.702491325
S8_13_1000	13	1000	5000	19.63365825	0.730854915
S8_13_400	13	400	5000	20.31794587	0.677120235
S8_19_1000	19	1000	5000	19.43925354	0.660604812
S8_19_400	19	400	5000	21.10614827	0.593231975
S16_13_1000	13	1000	10000	20.47029012	0.53196273
S16_13_400	13	400	10000	19.68169423	0.546690563
S16_19_1000	19	1000	10000	20.32534108	0.644834746
S16_19_400	19	400	10000	21.3179764	0.591457659
S8_13_1000	13	1000	10000	20.603892	0.624022237
S8_13_400	13	400	10000	19.01555554	0.700792445
S8_19_1000	19	1000	10000	19.75472265	0.632750533
S8_19_400	19	400	10000	19.22255874	0.676846355

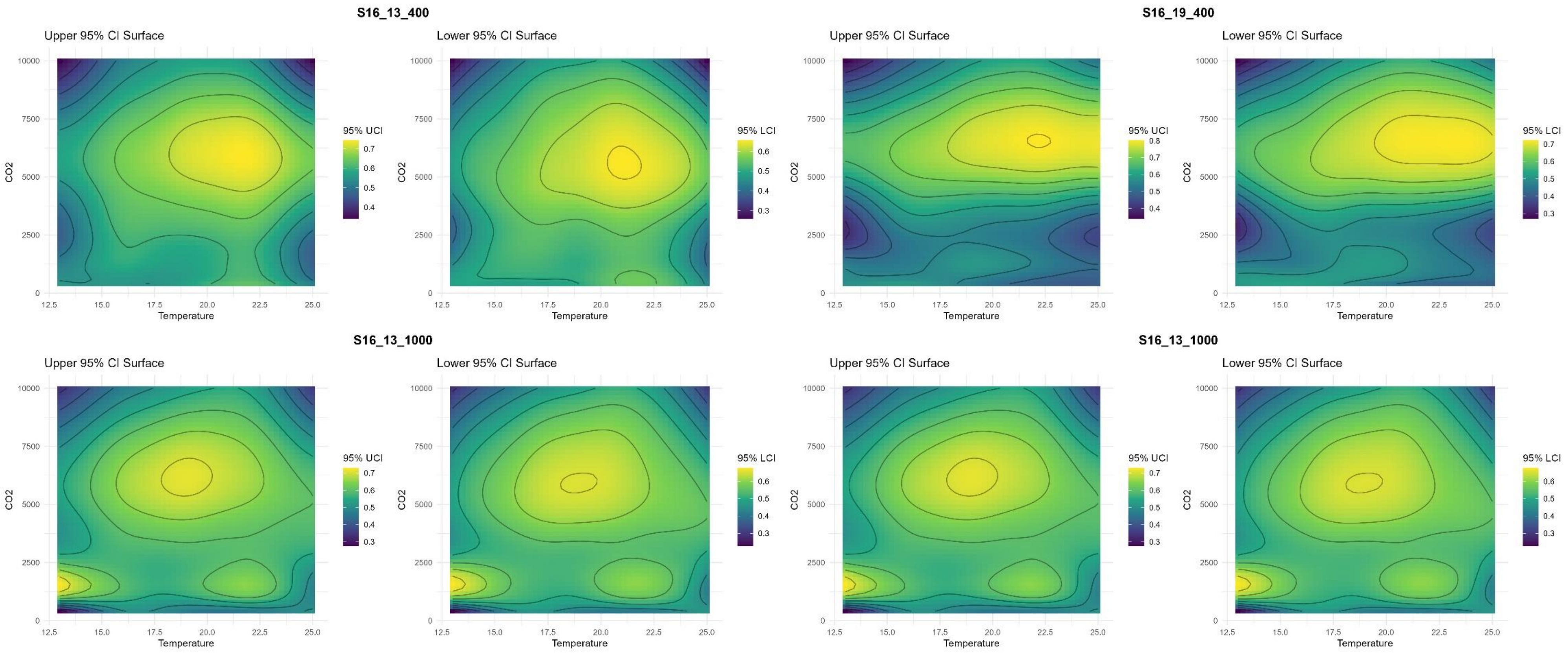

Figure S3: The upper and lower 95% confidence intervals (calculated using GAMs) for the temperature-CO_2 response surfaces (Figure 3) showing variation in growth rates across all temperature and CO_2 conditions for strain S16.

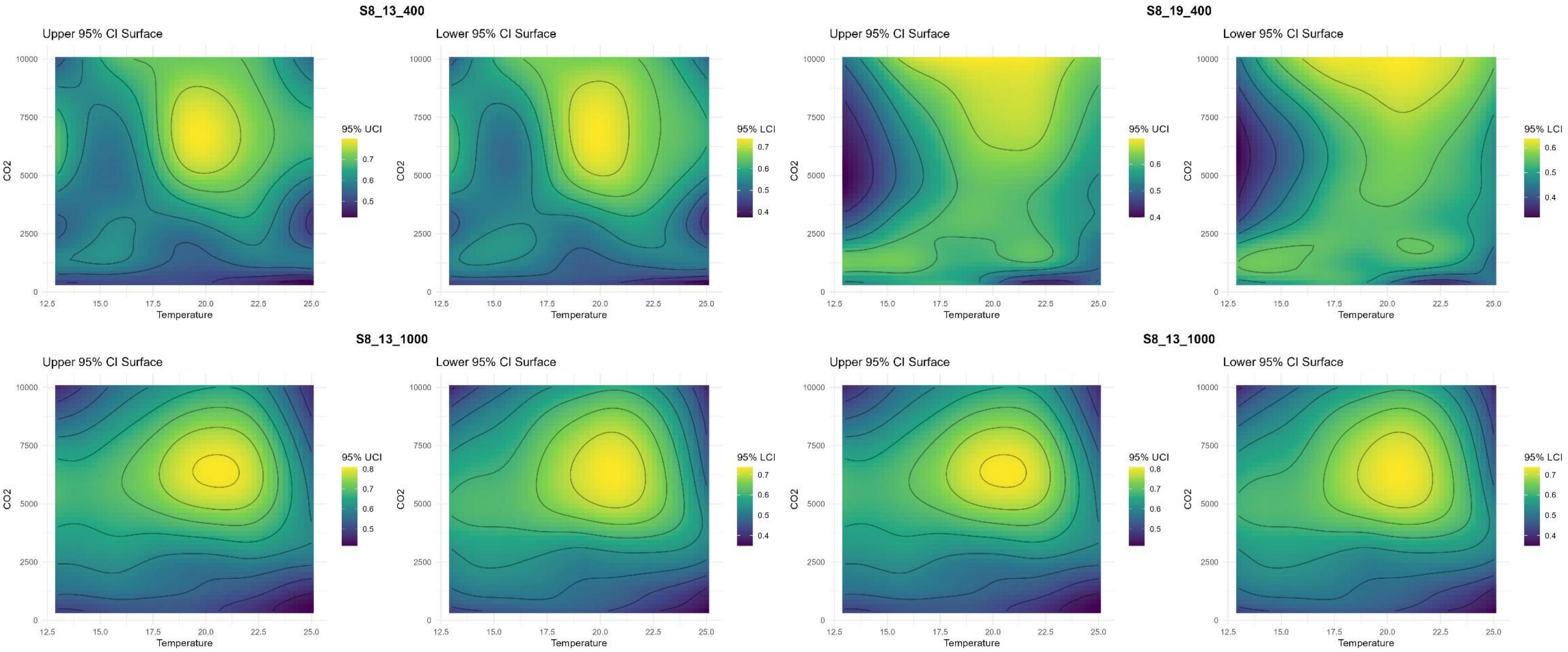

Figure S4: The upper and lower 95% confidence intervals (calculated using GAMs) for the temperature-CO_2 response surfaces (Figure 2) showing variation in growth rates across all temperature and CO_2 conditions for strain S8.

400 ppm

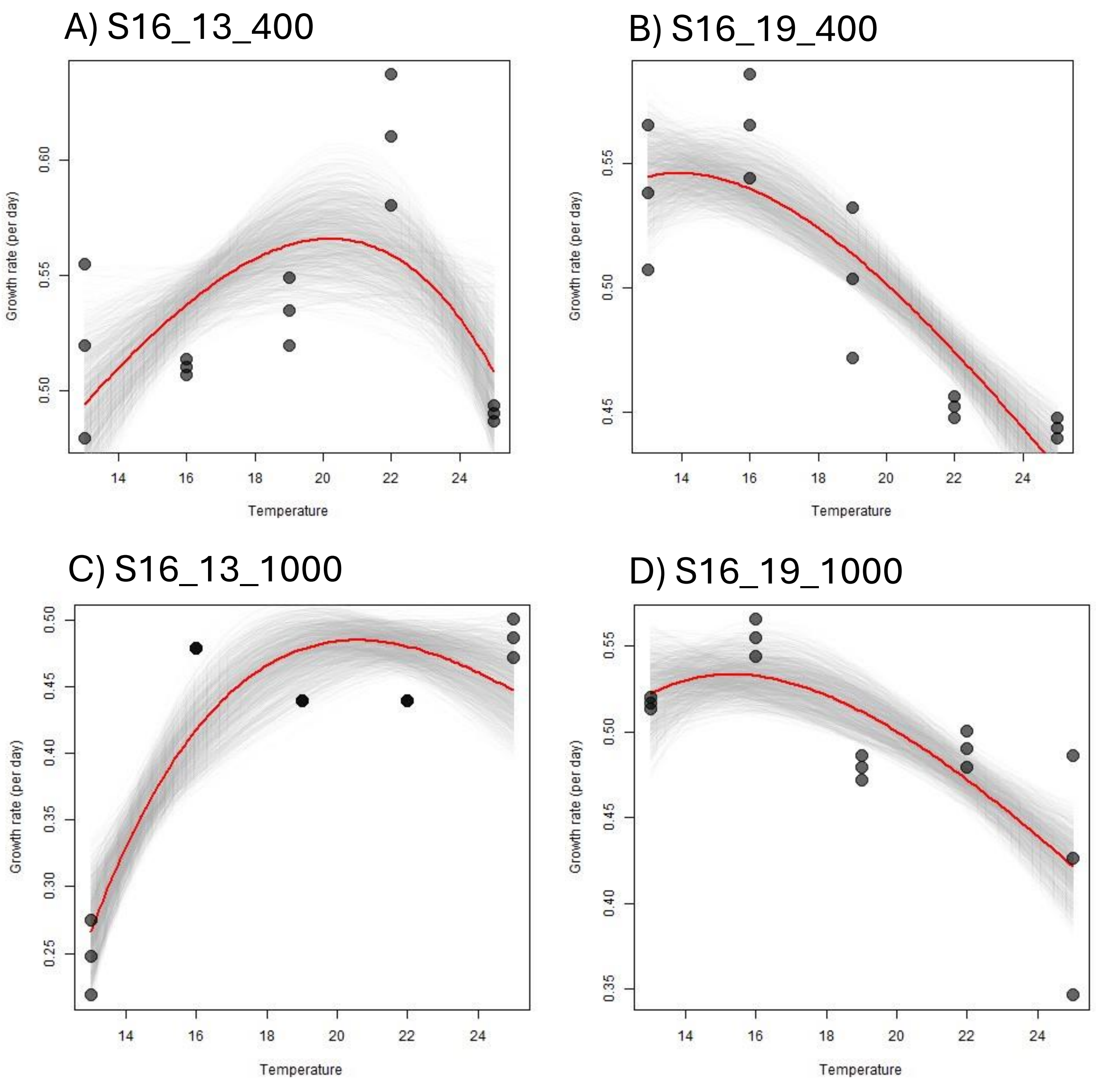

Figure S5a: Thermal performance curves for the strain S16 for all adaptation conditions for the 400 ppm CO_2 *treatment*

1000 ppm

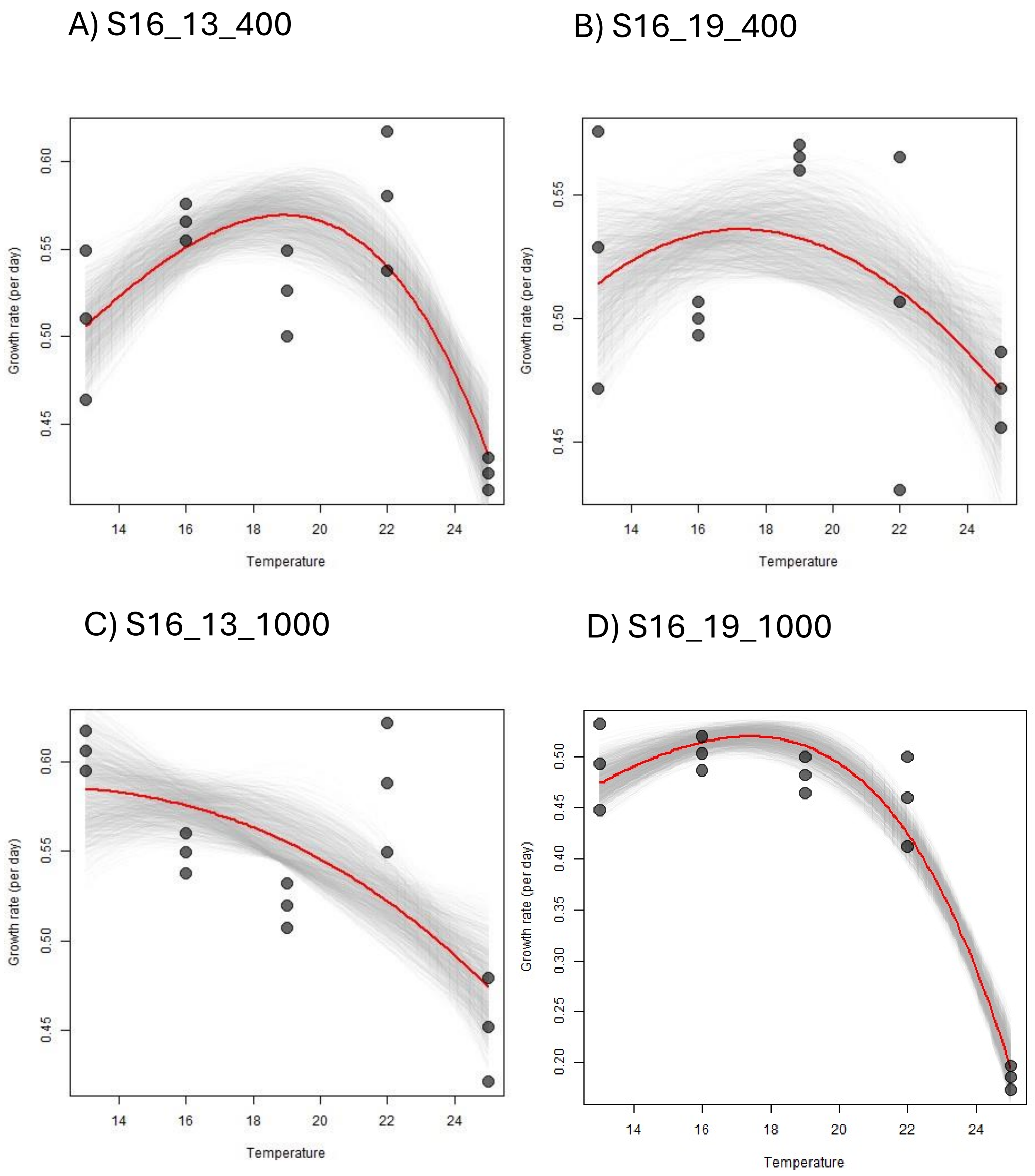

Figure S5b: Thermal performance curves for the strain S16 for all adaptation conditions for the 1000 ppm CO_2 treatment

2500 ppm

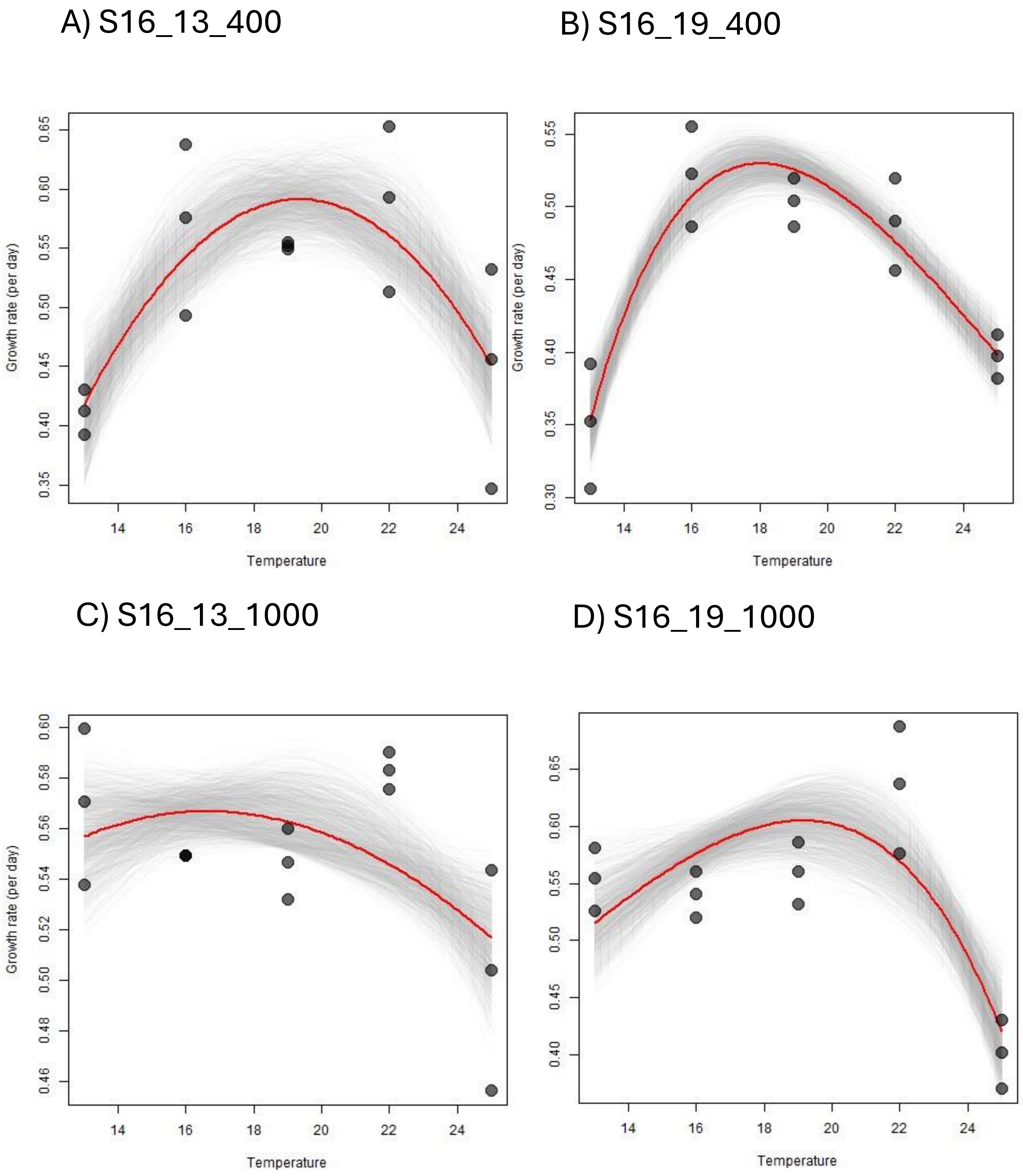

Figure S5c: Thermal performance curves for the strain S16 for all adaptation conditions for the 2500 ppm CO_2 treatment

5000 ppm

A) S16_13_400

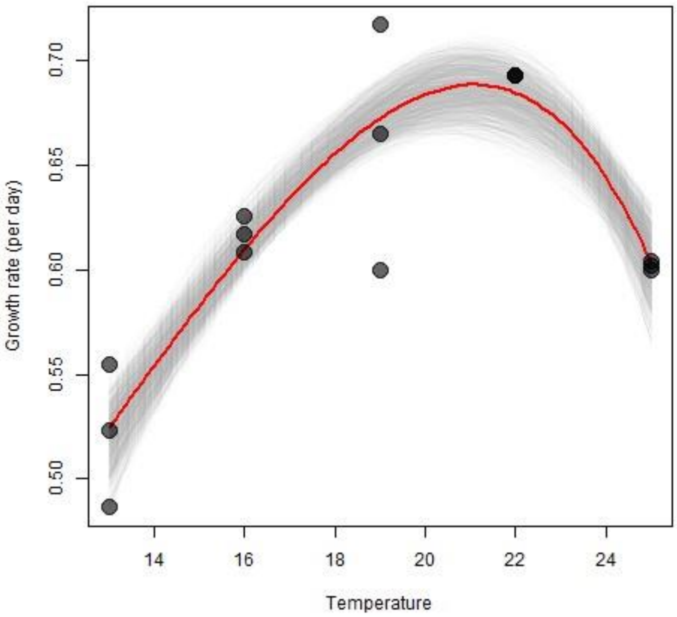

B) S16_19_400

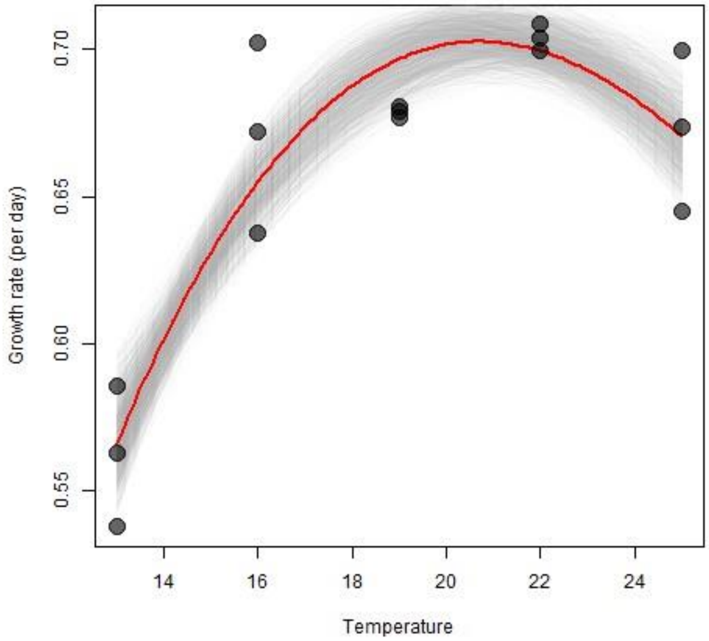

C) S16_13_1000

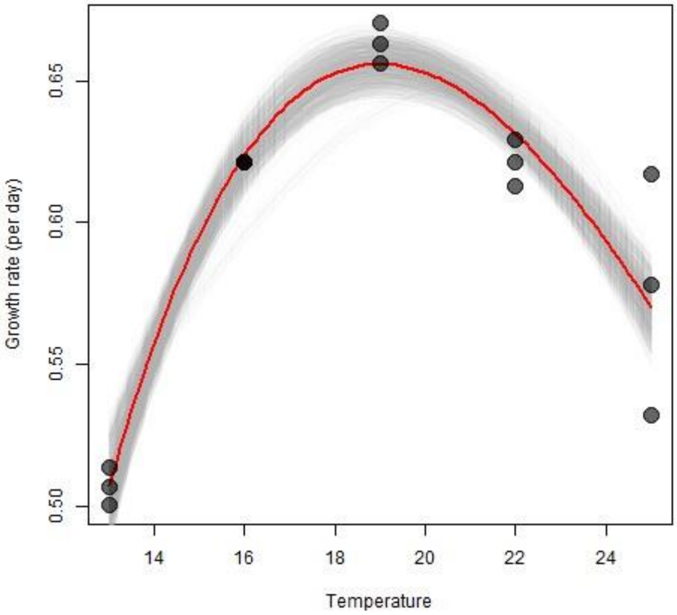

D) S16 19 1000

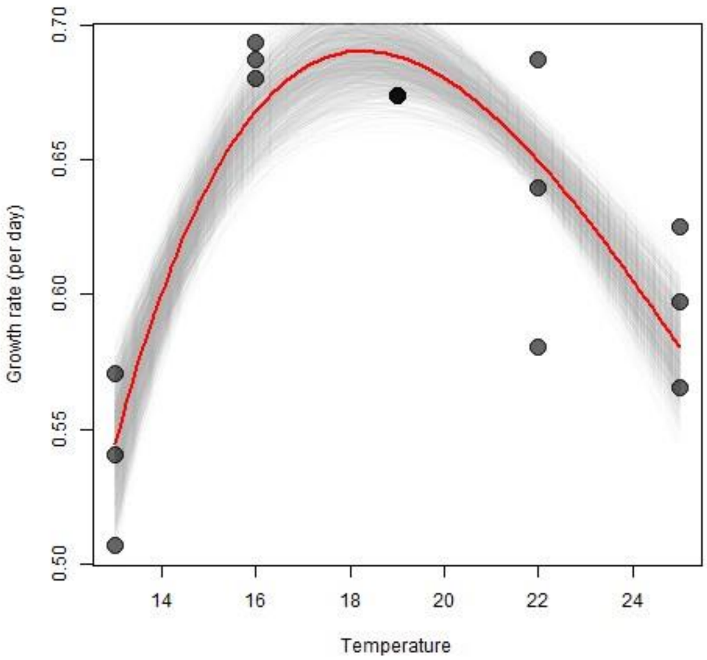

Figure S5d: Thermal performance curves for the strain S16 for all adaptation conditions for the 5000 ppm CO_2 treatment

10000 ppm

A) S16_13_400

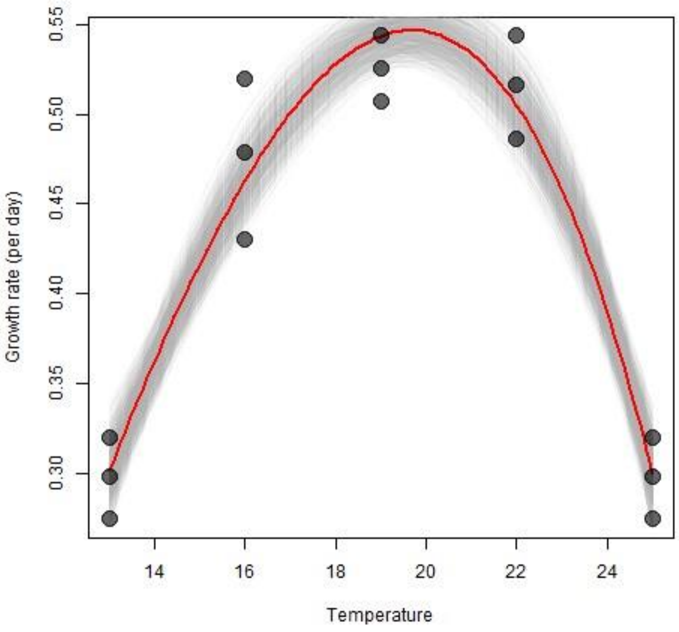

B) S16 19 400

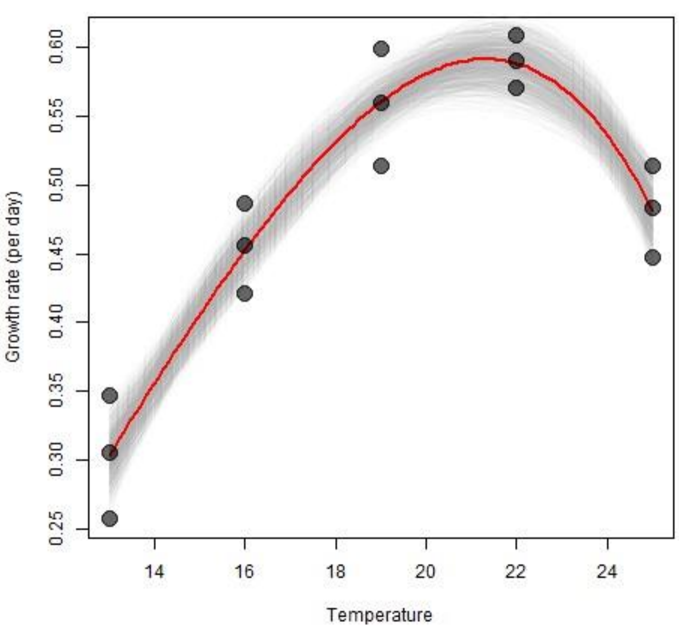

C) S16_13_1000

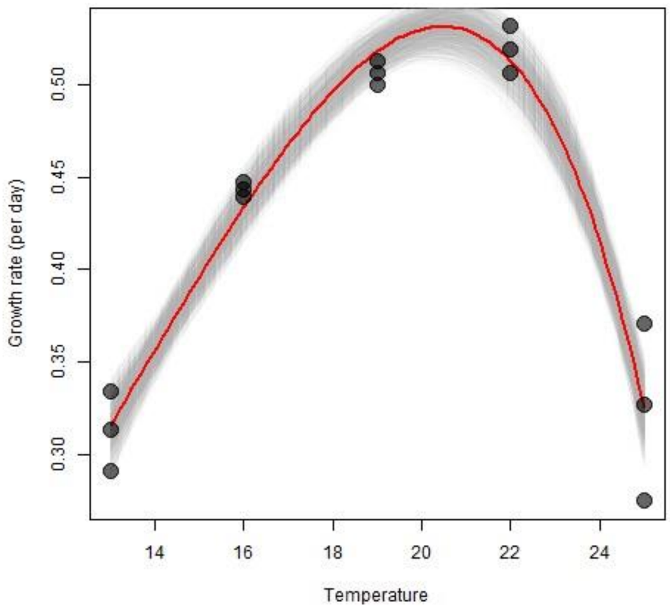

D) S16_19_1000

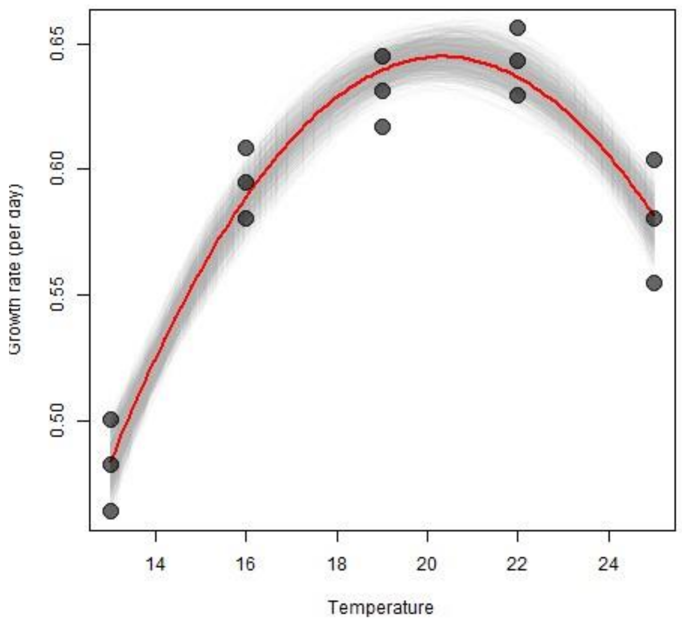

Figure S5e: Thermal performance curves for the strain S16 for all adaptation conditions for the 10000 ppm CO_2 treatment

400 ppm

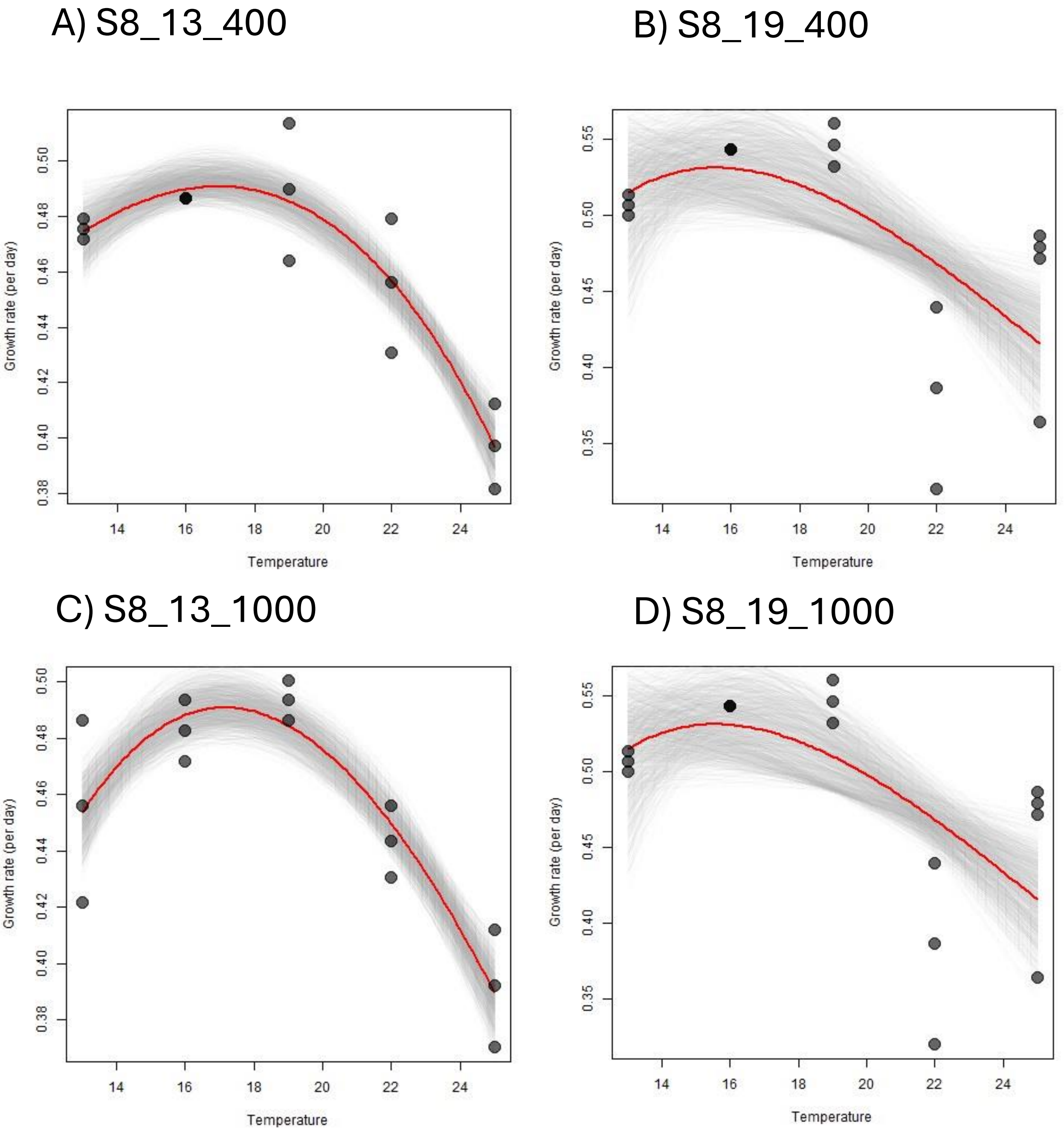

Figure S6a: Thermal performance curves for the strain S8 for all adaptation conditions for the 400 ppm CO_2 treatment

1000 ppm

A) S8_13_400

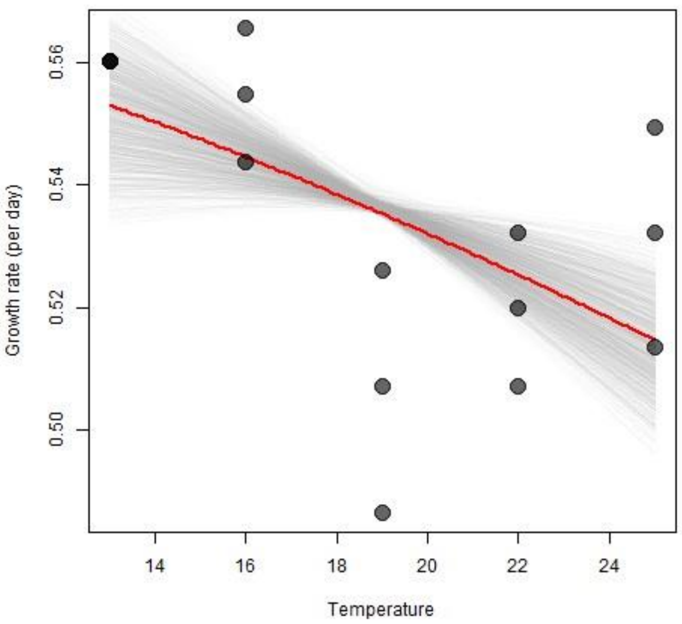

B) S8_19_400

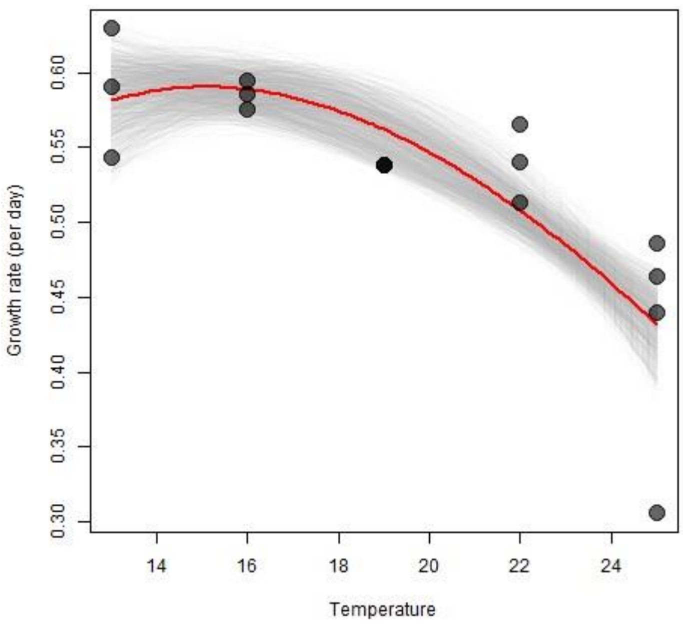

C) S8_13_1000

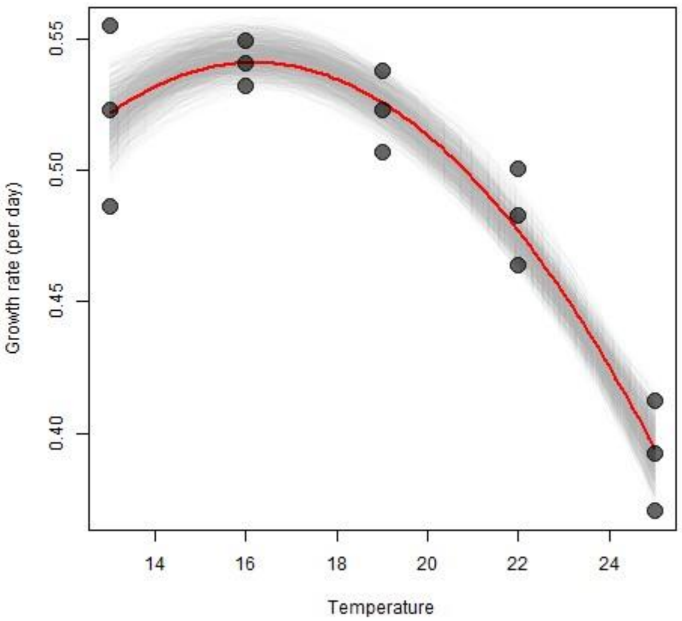

D) S8_19_1000

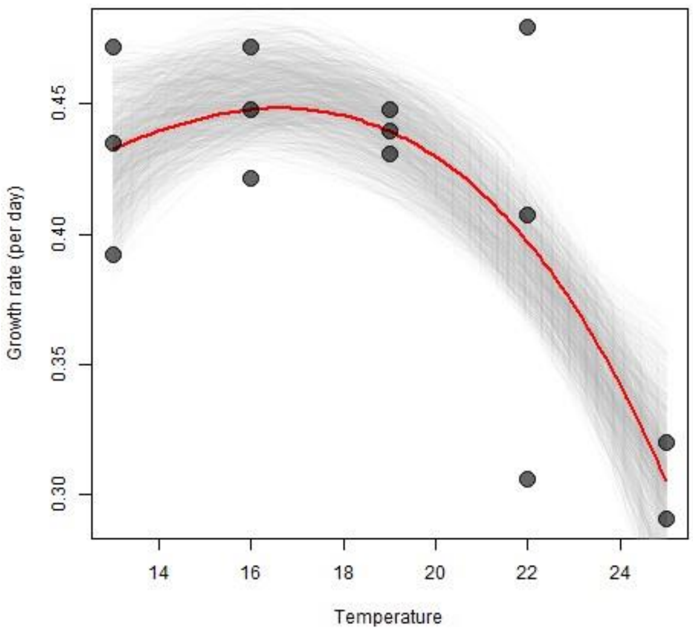

Figure S6b: Thermal performance curves for the strain S8 for all adaptation conditions for the 1000 ppm CO_2 treatment

2500 ppm

A) S8_13_400

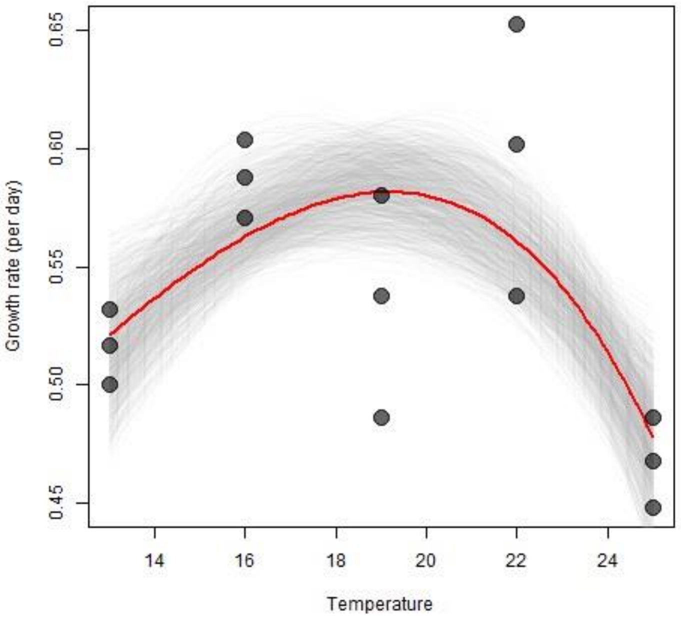

B) S8 19 400

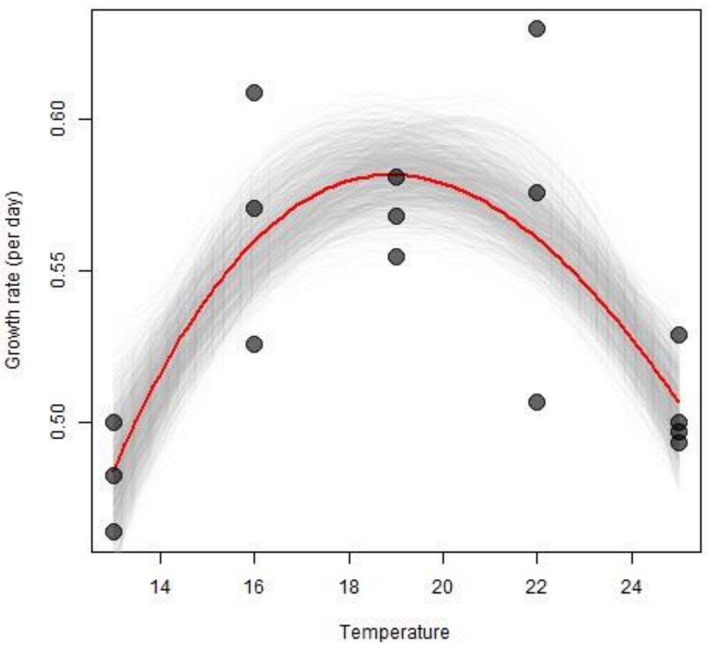

C) S8_13_1000

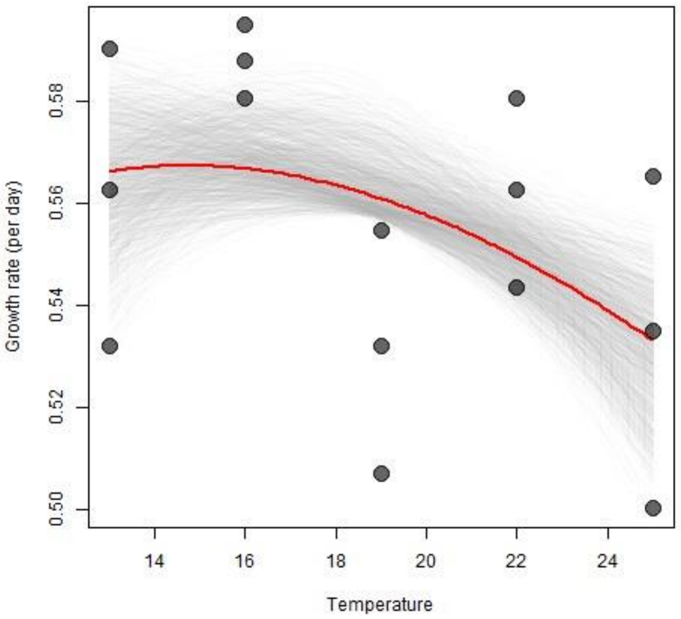

D) S8_19_1000

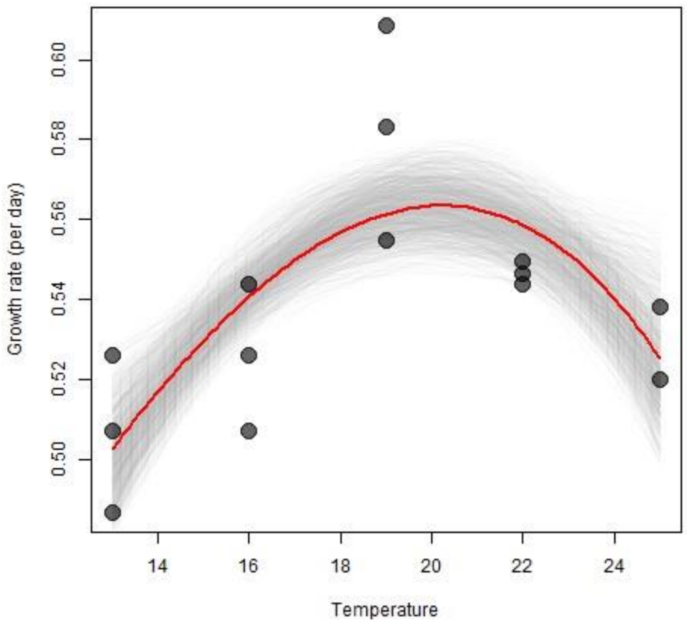

Figure S6c: Thermal performance curves for the strain S8 for all adaptation conditions for the 2500 ppm CO_2 treatment

5000 ppm

A) S8_13_400

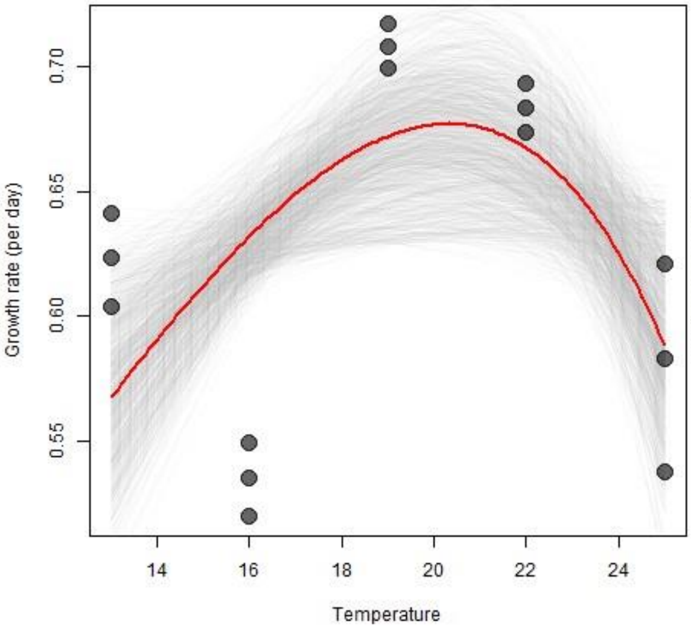

B) S8_19_400

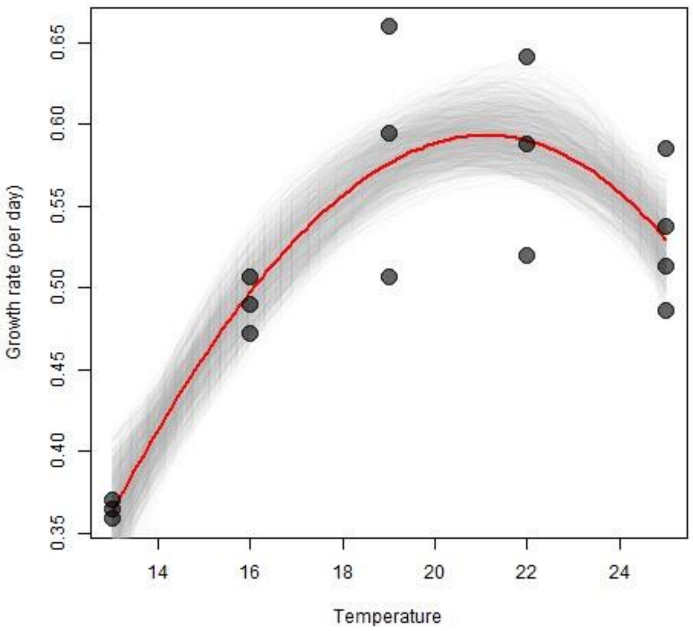

C) S8_13_1000

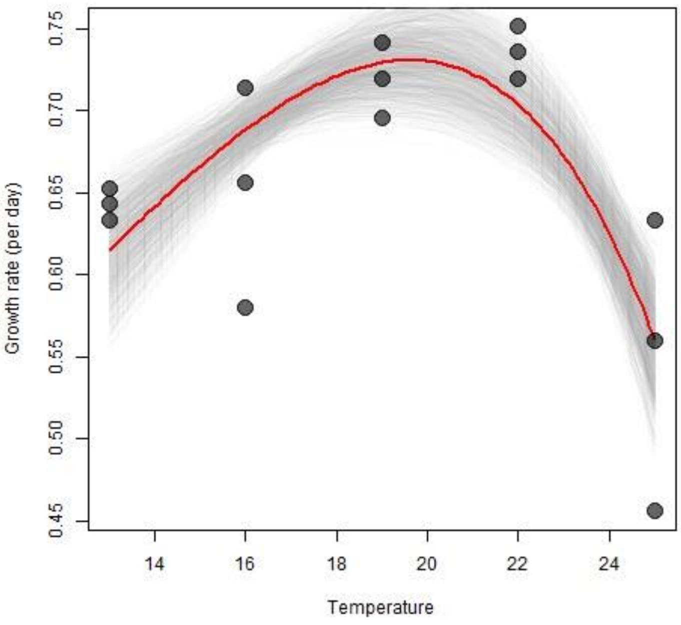

D) S8_19_1000

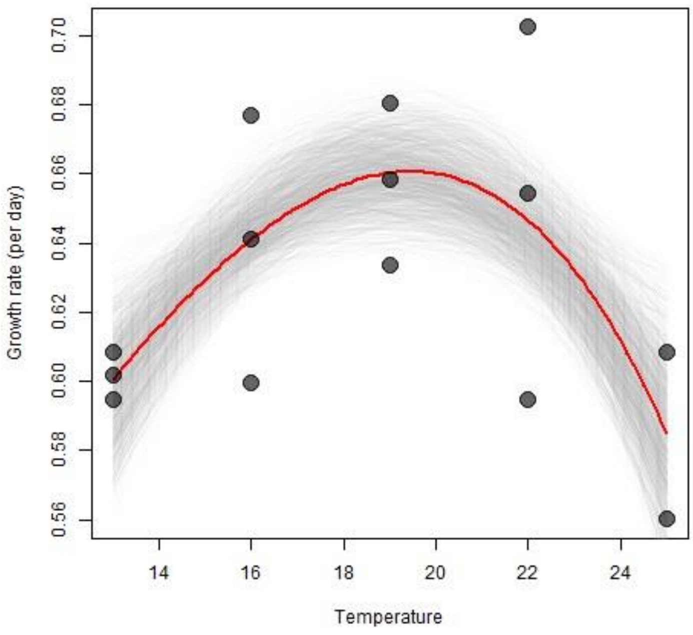

Figure S6d: Thermal performance curves for the strain S8 for all adaptation conditions for the 5000 ppm CO_2 treatment

10000 ppm

A) S8_13_400

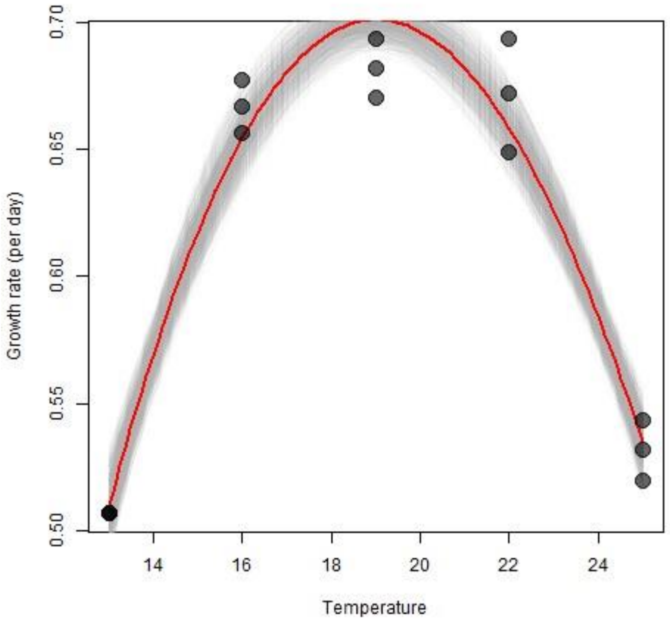

B) S8_19_400

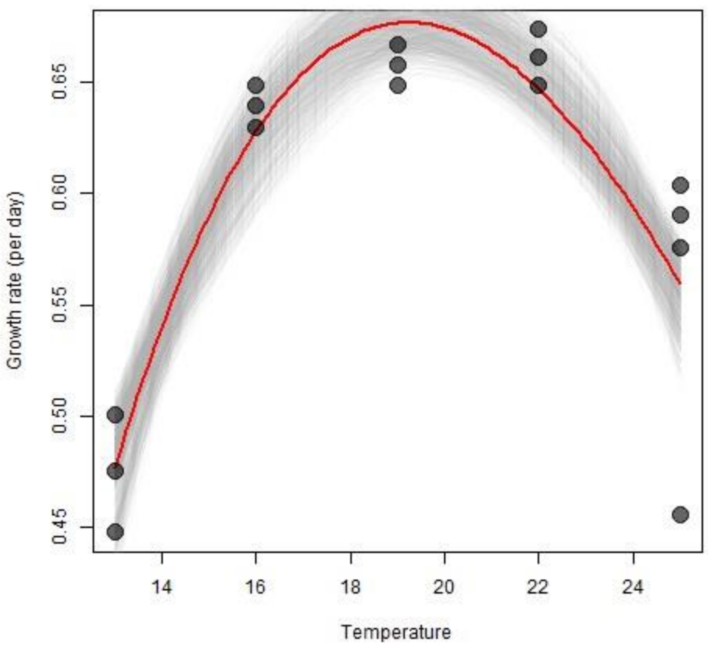

C) S8_13_1000

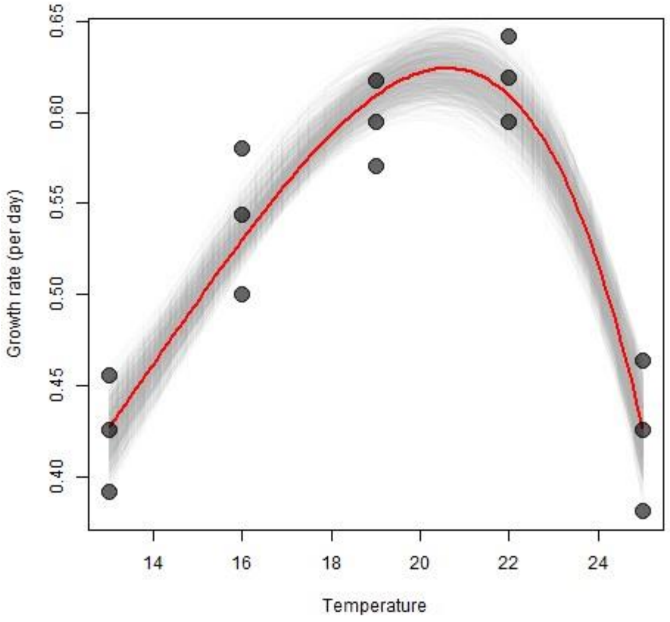

D) S8_19_1000

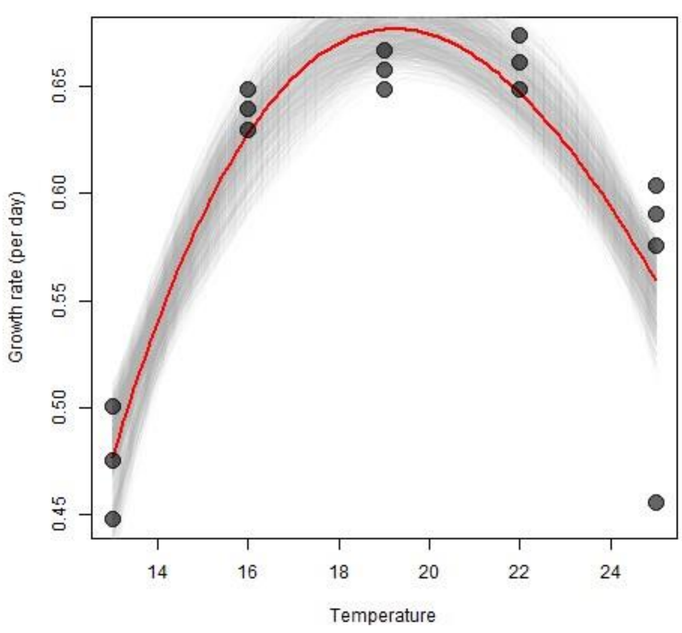

Figure S6e: Thermal performance curves for the strain S8 for all adaptation conditions for the 10000 ppm CO_2 treatment

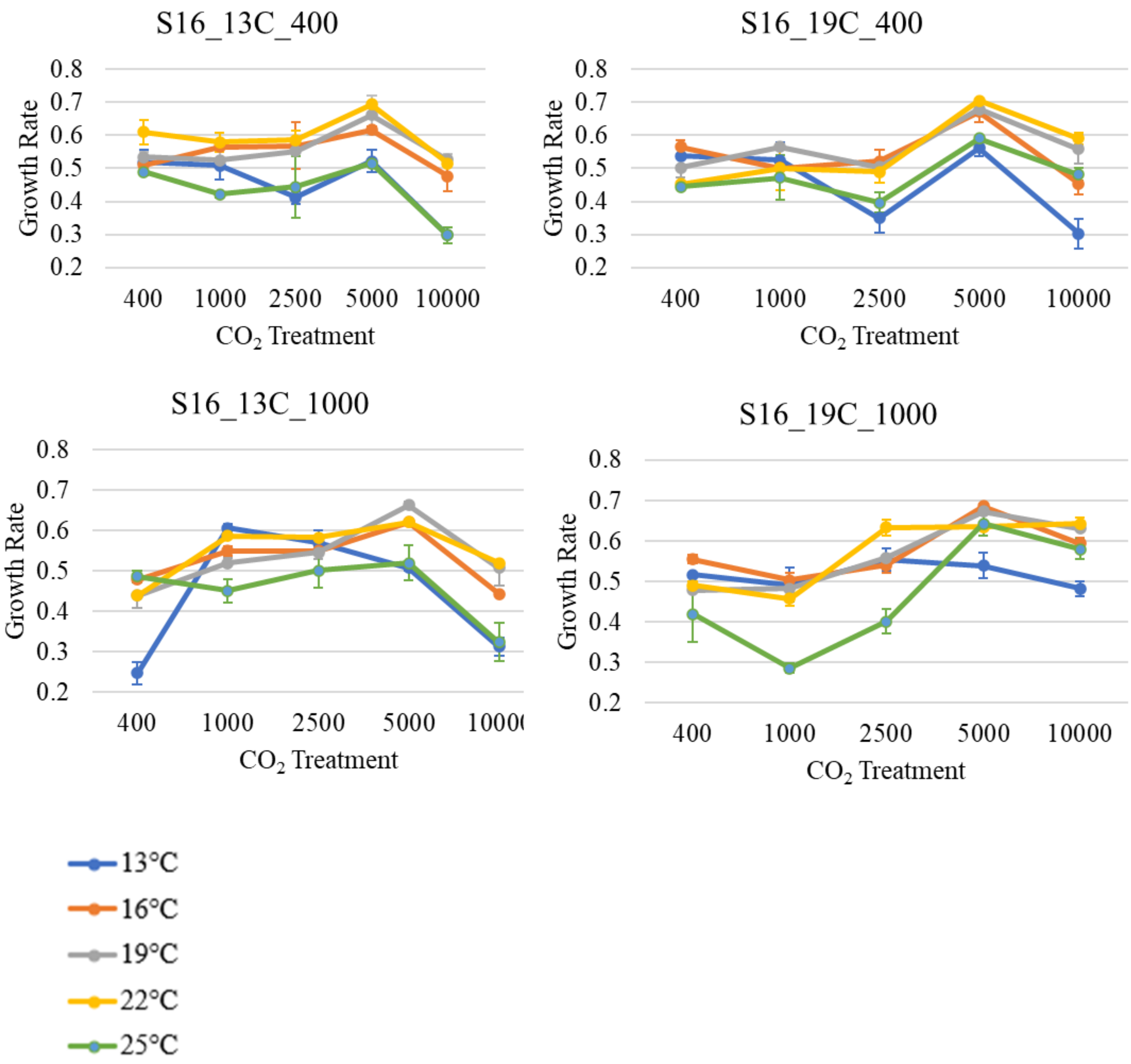

Figure S7a: The mean CO_2 curves for the adapted strain S16 for all five temperature conditions (13℃, 16℃, 19℃, 22℃ or 25℃).

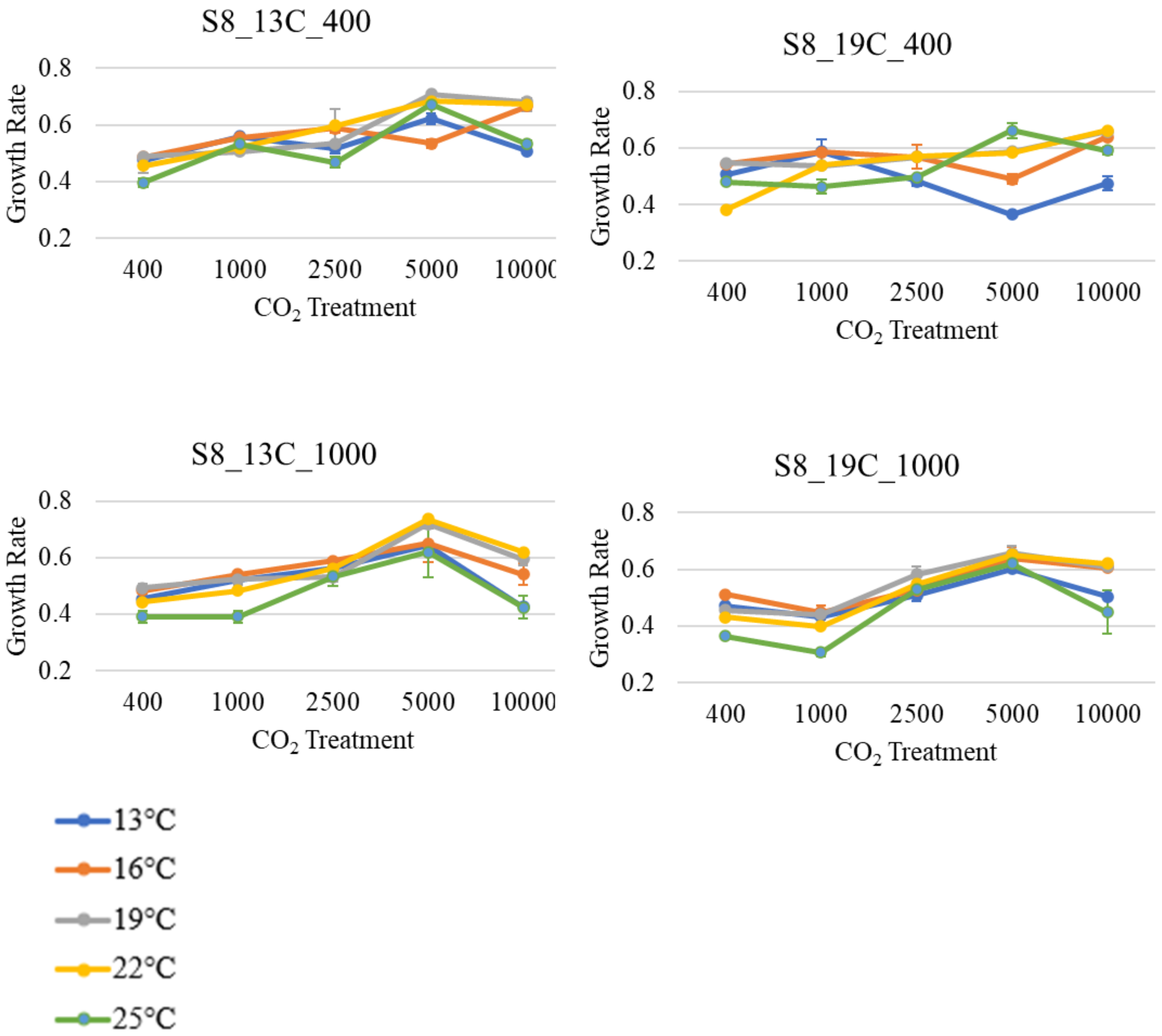

Figure S7b: The mean CO_2 curves for the adapted strain S8 for all five temperature conditions (13℃, 16℃, 19℃, 22℃ or 25℃).
